# Overlap of spike and ripple propagation onset predicts surgical outcome in epilepsy

**DOI:** 10.1002/acn3.52156

**Published:** 2024-10-07

**Authors:** Saeed Jahromi, Margherita A.G. Matarrese, Lorenzo Fabbri, Eleonora Tamilia, M. Scott Perry, Joseph R. Madsen, Jeffrey Bolton, Scellig S.D. Stone, Phillip L. Pearl, Christos Papadelis

**Affiliations:** ^1^ Neuroscience Research Center Jane and John Justin Institute for Mind Health, Cook Children's Health Care System Fort Worth Texas USA; ^2^ Department of Bioengineering The University of Texas at Arlington Arlington Texas USA; ^3^ Research Unit of Intelligent Health Technology for Health and Wellbeing, Department of Engineering Università Campus Bio‐Medico di Roma Rome Italy; ^4^ Fetal‐Neonatal Neuroimaging and Developmental Science Center Boston Children's Hospital, Harvard Medical School Boston Massachusetts USA; ^5^ Division of Epilepsy and Clinical Neurophysiology, Department of Neurology Boston Children's Hospital, Harvard Medical School Boston Massachusetts USA; ^6^ Division of Epilepsy Surgery, Department of Neurosurgery Boston Children's Hospital, Harvard Medical School Boston Massachusetts USA; ^7^ Burnett School of Medicine Texas Christian University Fort Worth Texas USA

## Abstract

**Objective:**

Interictal biomarkers are critical for identifying the epileptogenic focus. However, spikes and ripples lack specificity while fast ripples lack sensitivity. These biomarkers propagate from more epileptogenic onset to areas of spread. The pathophysiological mechanism of these propagations is elusive. Here, we examine zones where spikes and high frequency oscillations co‐occur (SHFO), the spatiotemporal propagations of spikes, ripples, and fast ripples, and evaluate the spike–ripple onset overlap (SRO) as an epilepsy biomarker.

**Methods:**

We retrospectively analyzed intracranial EEG data from 41 patients with drug‐resistant epilepsy. We mapped propagations of spikes, ripples, and fast ripples, and identified their onset and spread zones, as well as SHFO and SRO. We then estimated the SRO prognostic value in predicting surgical outcome and compared it to onset and spread zones of spike, ripple, and fast ripple propagations, and SHFO.

**Results:**

We detected spikes and ripples in all patients and fast ripples in 12 patients (29%). We observed spike and ripple propagations in 40 (98%) patients. Spike and ripple onsets overlapped in 35 (85%) patients. In good outcome patients, SRO showed higher specificity and precision (*p <* 0.05) in predicting resection compared to onset and zones of spikes, ripples, and SHFO. Only SRO resection predicted outcome (*p* = 0.01) with positive and negative predictive values of 82% and 57%, respectively.

**Interpretation:**

SRO is a specific and precise biomarker of the epileptogenic zone whose removal predicts outcome. SRO is present in most patients with drug‐resistant epilepsy. Such a biomarker may reduce prolonged intracranial monitoring and improve outcome.

## Introduction

For patients with drug‐resistant epilepsy (DRE), the best available treatment is surgery, achieving seizure freedom in ~60% of cases.^1^ The epilepsy surgery outcome depends upon the epileptogenic zone (EZ) delineation.^2^, ^3^ Due to lack of methods that measure the EZ directly, this area is defined indirectly based on data from several noninvasive tests.^4^, ^5^, ^6^ However, the results of these tests are often non‐concordant. Intracranial electroencephalography (iEEG) serves as the gold standard for defining the seizure onset zone (SOZ), the EZ's most logical approximator. However, the SOZ does not always predict outcome^7^ while its delineation requires several days of recordings at the expense of human and financial resources. Thus, there is an ultimate need for a novel interictal EZ biomarker.

Spikes are the primary interictal epilepsy biomarkers. Spikes networks correlate with surgical outcome.^8^, ^9^, ^10^ Yet, spikes are limited in localizing the EZ, as they are also found in physiological areas.^11^, ^12^ High‐frequency oscillations (HFOs), categorized into ripples (80–250 Hz) and fast ripples (250–500 Hz), seem to be more specific interictal biomarkers.^4^, ^13^, ^14^ Ripples often overlap with hubs that generate ictal ripples during seizures.^15^, ^16^, ^17^ However, their clinical value is limited since they also occur in physiological regions.^18^ Previous iEEG studies show that ripples co‐occurring with spikes are marginally better,^19^ or of equal effectiveness,^20^ in predicting the SOZ compared to all spikes. Fast ripples are more specific biomarkers of the EZ^21^; their resection shows the highest outcome predictive value and longest time until the first postoperative seizure.^14^ However, their resection does not guarantee seizure freedom.^22^ Moreover, fast ripples are challenging to record using macroelectrodes^23^ limiting their utilization to few patients.^20^

Similar to seizures, interictal biomarkers propagate across multiple contacts^24^, ^25^, ^26^ from onset to spread areas.^27^, ^28^, ^29^ Spikes show early peaks which travel to later propagated activity.^25^, ^29^ These spike waves traverse to both adjacent and distant brain areas^30^ following the same path and direction as ictal discharges.^31^ Regions where discharges show the earliest peaks are located inside the EZ, whereas sites with late propagated activity are less epileptogenic.^6^, ^9^, ^26^, ^27^ Previous studies have shown that spike onset correlates with the SOZ while its removal is associated with good outcome.^9^, ^27^ Contrarily, other studies have shown that spike onset is not related to SOZ^30^ and its resection is not superior to resection of high spike rate iEEG channels.^10^ Similarly, ripples propagate across iEEG contacts.^26^ Ripple onset resection is associated with good outcome though is not superior to resecting channels with high HFO rates.^32^ The pathophysiological mechanism of these propagations and the spatial relationship among them is unclear. Moreover, it is unknown whether these propagation onsets overlap and whether this overlapping onset serves as a more specific EZ biomarker compared to individual onsets.

This study aims to map the spatiotemporal propagations of spikes, ripples, and fast ripples, examine their temporal and spatial features, and evaluate the clinical significance of the spike–ripple onset (SRO) overlap (or intersection) as an interictal biomarker of the EZ. We hypothesize that the SRO's specificity in delineating the EZ and predicting outcome surpasses that of individual onsets and the clinically defined SOZ.

## Material and Methods

### Cohort

We retrospectively analyzed iEEG data from patients with DRE who had brain surgery at Boston Children's Hospital between 2011 and 2018. The inclusion criteria were (i) postsurgical follow‐up of at least 1 year; (ii) long‐term monitoring using electrocorticography and/or stereotactic EEG; and (iii) availability of pre‐ and postsurgical MRI. The study was approved by North Texas Regional IRB. Informed consent was not required due to the study's retrospective nature.

### iEEG data acquisition

Long‐term monitoring was performed for several days using subdural grids and/or strips (2–3 mm diameter, 10 mm inter‐electrode distance, Ad‐Tech., USA) and/or depth electrodes (6–16 contacts, 0.8 mm diameter, 3–5 mm inter‐electrode distance, Ad‐Tech. or PMT, USA). Data were recorded using XLTEK Quantum NeuroWorks (Natus Inc., USA) with frequencies between 1000 and 2048 Hz (Table 1). Positions of implanted electrodes were defined using an in‐house co‐registration procedure^33^; electrodes within the white matter were excluded. Video recordings were performed simultaneously, and segments were chosen after visual inspection to ensure no apparent REM activity.^34^ We selected 5‐min epochs from non‐REM slow wave sleep, when feasible, given the high spikes and HFOs rates^35^, ^36^ and minimal motion artifacts during this stage.

**Table 1:** Patients demographic and clinical information.

ID/sex	Age^a^, [years]	Epilepsy onset, years	MRI findings	Implantation side	Res. region	iEEG (SE + DE)	Spike prop. [no.]	Ripple prop. [no.]	FR prop. [no.]	Engel (f/u, [years])	Sam. freq. [Hz]
1/M^†^	11	4	Normal	R	Fr	80 (SE)	162	131	79	IA (5)	2000
2/M	10	2	Normal	L	IH, CG	88 + 10	5	11	0	IA (4)	2000
3/F^†^	7	3	FCD (T and Ins)	L	Fr, T	90 (DE)	9	11	0	IB (8)	2048
4/F	14	10	Normal	L	T	72 (SE)	2	9	0	IA (5)	2000
5/M	18	9	Tumor (T)	L	T	72 + 20	319	67	0	IC (6)	2000
6/F	16	14.5	FCD (T)	L	T, Hipp.	96 + 10	52	38	0	IA (8)	2000
7/M^†^	2	0.3	TSC (multifocal)	R	Fr	112 (SE)	84	65	0	IA (7)	2000
8/F^†^	9	4	FCD (P)	R	P	80 + 20	103	87	0	IB (4)	2000
9/M	2	0	FCD (CF)	L	Fr, P	80 (SE)	28	5	0	IA (6)	2000
10/M^¥^	18	17	Parahippocampal (L)	L	T	64 (SE)	1	1	0	IA (2)	2000
11/F	18	15	Normal	L	T	88 (SE)	2	24	0	IA (7)	2000
12/M	15	12	HS (AT)	L	T	80 (SE)	2	52	0	IA (2)	2000
13/F	15	6	FCD (mesial P)	L	Fr	72 (SE)	460	58	0	IA (2)	2048
14/M^†^	5	0.17	FCD (T)	L	T, O	96 (SE)	176	15	8	IA (2)	2000
15/F^¥^	3	1.33	TSC (multifocal)	L	Fr	120 (SE)	22	3	0	IA (2)	2000
16/M	4	1	FCD (Fr)	L	Fr	56 + 10	10	13	0	IC (6)	2048
17/M^†^	22	5	FCD (CP)	L	Fr	64 + 30	30	21	4	IA (3)	2048
18/F^¥^	12	1.17	Normal (pineal cyst)	L	Fr	70 (DE)	163	0	0	IA (4)	2000
19/M^†^	10	7	PMG (Fr, P)	L	Fr	64 + 60	73	52	0	IB (2)	2000
20/M^†^	4	2	FCD (Fr)	R	Fr	128 + 10	174	307	39	IA (2)	2048
21/F	16	2	FCD (L, Fr)	L	Fr	72 + 40	1	32	0	IA (1)	2048
22/F	6	3	Lesion, CTH (P)	L	Fr, P	80 + 20	5	28	0	IA (1)	2048
23/F^†^	6	4	PMG (P, T, O)	R	P, O	196 (DE)	118	117	23	IA (1)	2048
24/M	14	5	Normal	L	O, P, T	236 (DE)	8	57	0	1A (1)	2048
25/M^¥^	12	0.5	FCD (L, Fr)	L	P, T	72 + 24	9	6	0	1A (2)	2048
26/M	19	12	Normal	L	Fr, T, P	140 (SE)	19	60	0	IIA (1)	1028
27/F^†^	10	0.3	HS (MeT, periventricular)	L	T	140 (DE)	63	31	25	IVB (6)	2048
28/M^†^	6	2	FCD (Fr)	L	Fr	120 (SE)	382	385	201	IIIA (11)	2000
29/M	16	4	Normal	L	T	88 (SE)	16	11	0	IIB (2)	2000
30/M	16	4	Normal (mild gliosis)	L	Fr	88 (SE)	985	1	0	IIIA (5)	2000
31/F	5	0.5	TSC (P, O)	L	O	128 + 40	76	93	0	IIIA (2)	1024
32/F	13	7	Normal	R	Fr	112 + 10	4	15	0	III (1)	2000
33/M	17	1.5	Glioma (PO junction)	R	O, P, T	128 (SE)	1	21	0	IIIA (4)	1000
34/F	18	4	FCD (Fr)	L	Fr	144 + 10	96	15	0	IIA (1)	1028
35/M^†^	13	8	Encephalomalacia (LP, SUP T Lobe)	L	Fr, P	64 + 30	142	51	0	IIB (1)	2000
36/M	13	7	FCD (T)	L	Fr, T	112 + 10	2	7	0	IIIA (6)	2048
37/M	13	0	Infarct (MCA territory)	L	Fr, T	136 (SE)	440	279	0	IIA (3)	1024
38/M	18	5	FCD (SUP Fr gyr.)	L	Insula	212 (DE)	40	10	0	IIIA (6)	2048
39 /F^¥^	22	14	Trauma	R	F, T	120 (SE)	51	8	0	IIB (5)	2000
40/F^¥^	7	4	FCD (Fr operculum)	R	Fr	72 + 40	0	25	0	IIA (3)	1000
41/M	10	5	Normal	L	Fr	96 + 10	117	13	0	III (1)	2048

A, anterior; CG, cingulate gyrus; CTH: cortical thinning; DE, depth electrodes; F, female; FCD, focal cortical dysplasia; Fr, frontal; gyr., gyrus; HS, hippocampal sclerosis; L, left; M, male; Me, mesial, O, occipital; P, parietal; PMG, polymicrogyria; Prop., propagation; R, right; Res., resected; SE, subdural electrodes; Sam. Freq., sampling frequency; SUP, superior; T, temporal; TSC, tuberous sclerosis complex.

^a^ Age = age at epilepsy surgery.

^¥^ Patient with no overlap in onsets of spike and ripple propagations.

^†^ Patient has fast ripples.

### Resection and outcome

The resection was defined by co‐registering pre‐ and postsurgical MRIs. Each electrode was regarded as *resected* if it was inside or within 10 mm from resection and *non‐resected* otherwise.^6^, ^37^, ^38^, ^39^ The outcome was assessed during a follow‐up visit by an epileptologist (J.B.) using the Engel scoring system.^40^ Patients were dichotomized into good (Engel I) or poor (Engel ≥II) outcome.

### Spikes and HFOs detection and Co‐occurrence

The data were preprocessed by applying a DC offset removal, a notch filter (60 Hz) and its harmonics. Signals were then bandpass filtered between 1 and 70 Hz for spikes, 80 and 250 Hz for ripples, and 250 and 500 Hz for fast ripples. Spikes were detected using *Persyst 14.0* (Persyst Development Co., CA)^41^ which was validated against readings of an EEG expert (C.P.).^9^ HFOs were initially detected automatically using *RippleLab* ^42^ and were further validated visually (Supplementary Material on Spike and HFO detection and Table S1). We defined the co‐occurring spike and HFO (SHFO) zones as electrodes containing spikes with HFOs occurring within a 50 ms window around them.^43^

### Spike and HFO propagations

**Figure 1:**
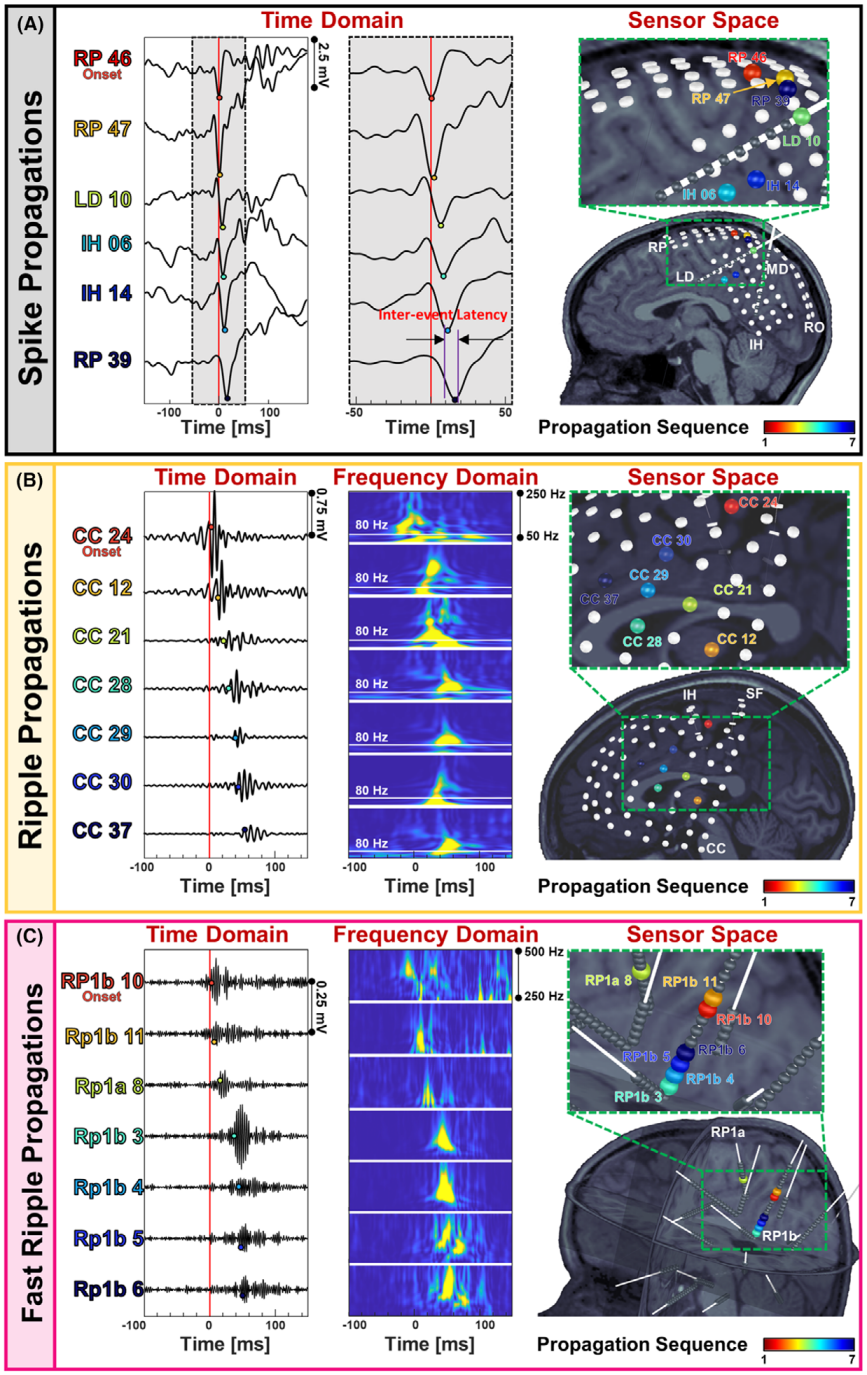
Sequences of spike, ripple, and fast ripple propagations. (A) Spike propagation in time domain (ms) across depth electrodes implanted in a 9‐year‐old female patient with drug‐resistant epilepsy (DRE) (patient #8). The first event in propagation is considered as the onset event, followed by the next events. The time window between two successive events (i.e., *inter‐event interval*) in a spike propagation was set to 10 ms. Electrodes that participate in propagation are color‐coded according to their order in propagation. The propagation sequence is shown color‐coded on electrodes co‐registered on patient's MRI, with the onset electrode colored in red and the last electrode in the propagation colored in blue. (B) Ripple propagation in time domain (ms) across subdural electrodes implanted on the cortex of a 11‐year‐old male patient with DRE (patient #1). The *inter‐event interval* for ripple propagations was set to 30 ms. Time‐frequency plots of each of the events in propagation are also displayed (50–250 Hz); the 80 Hz frequency is indicated to define the lower boundaries of the ripple frequency band. The sequence of this propagation is showed on patient's MRI. The electrodes that participate in the propagation are colored according to the order they appear in propagation. (C) Fast ripple propagation in time domain (ms) across depth electrodes implanted in a 6‐year‐old female patient (patient #23). The *inter‐event interval* for fast ripple propagations was set to 15 ms. The time‐frequency plot of each of the events in the propagation is plotted (250–500 Hz). The propagation sequence is shown color‐coded on electrodes co‐registered on patient's MRI. The electrodes that participate in the propagation are colored according to the order they appear in the propagation.

We developed an in‐house algorithm that identifies propagation sequences of spikes (Fig. 1A), ripples (Fig. 1B), and fast ripples (Fig. 1C). The algorithm sorts spike, ripple, and fast ripple events in time and marks the earliest event within each propagation as onset. Then, it adds the next event of the same type to the sequence if it occurred within a specific time window, defined as *inter‐event latency* (Fig. 1A). Inter‐event latency for spikes and ripples are within the range of 3–15 ms^10^, ^26^ and 10–32 ms,^6^, ^26^ respectively. Ripples have higher latencies due to pyramidal cell synchronization; unlike spikes, which arise from imbalances in inhibitory and excitatory neurotransmission. We set the *inter‐event latency* to 10, 30, and 15 ms, for mapping spike, ripple, and fast ripple propagations, respectively.^6^, ^10^, ^26^ No previous studies report inter‐event latency for fast ripples. Finally, we discarded propagations having >50% of their events occurring within 2 ms from each other.^44^, ^45^

### Electrodes ranking

Each electrode acquired a normalized electrode rank (ER) based on its temporal activity in propagations (Supplementary Material on Electrodes Ranking). We computed the normalized *ER* separately for spike, ripple, and fast ripple propagations for each patient, that is, electrodes with higher normalized ranks contain events that contribute to propagations' onset (*onset electrodes*), whereas electrodes with lower normalized ranks contribute to propagations' spread (*spread electrodes*).

### Definition of onset, spread, and spike–ripple overlap zones

We used the normalized *ER* to estimate *onset thresholds* for categorizing iEEG electrodes into *onset* and *spread* electrodes for each propagation type. We considered resected electrodes in each good outcome patient as ground truth for defining the EZ. We varied the *ER* threshold between 0 and 100% (5% steps) for each patient to predict resected electrodes. An electrode with a normalized rank above threshold was considered as true‐positive (TP) if the electrode was resected and as false‐positive (FP) if not resected. An electrode with a normalized rank below the threshold was considered as true‐negative (TN) if the electrode was not resected and as false‐negative (FN) if resected. For each threshold, we calculated sensitivity [TP/(TP + FN)], specificity [TN/(TN + FP)], and Youden's index (J) (sensitivity + specificity − 1) and selected the optimum threshold as the one yielding the maximum J for that patient. Finally, we calculated the onset threshold as the average of optimum thresholds over good outcome patients for each propagation type and defined three zones for spike, ripple, and fast ripple propagations: (i) onset zone, that is, electrodes with normalized ranks above the onset threshold; (ii) spread zone, that is, electrodes with normalized ranks equal to or below the onset threshold; and (iii) SRO zone as electrodes that contribute to both onsets of spike and ripple propagations. Also, electrodes containing spikes, ripples, and fast ripples were defined as entire zones of these biomarkers.

### Temporal and spatial propagation features

We defined propagation rates (No./s) as the number of events over the segment duration and computed the proportion of events participating in propagations. We defined the propagation duration (ms) as the latency between onsets of the first and last events in propagation, and latency variability (ms) as the time difference between consecutive events in a propagation. We measured propagation displacement (cm), as the sum of Euclidian distances between consecutive electrodes in the propagation, and displacement variability (cm) as the Euclidian distance between two consecutive electrodes in the propagation. We also calculated propagation velocity (m/s) as the distance traveled by the propagation over its duration, and velocity variability (m/s) as the Euclidean distance between two consecutive electrodes in a propagation over the time latency between their corresponding events, for each patient. Finally, we extracted the median number of channels that participated in each type of propagation per patient.

### Distance from resection, resection ratio, and onset variability

We defined the resection ratio of each zone for each patient as the percentage of resected electrodes in that zone. Moreover, we calculated the average distance (mm) of each zone from resection. Finally, we assessed for each patient the spatial variability of onset zones for each propagation type, namely the “onset variability,” as the average of minimum distances (mm) between onset electrodes of each propagation and onset electrodes of other propagations of the same type. We also quantified the concordance of SRO and SOZ zones in each patient by calculating the distance of SRO electrodes from SOZ electrodes and estimating the percentage of SRO electrodes which overlapped with SOZ.

### Outcome prediction

We computed outcome predictability for SHFO, SOZ, SRO, propagation onset and spread, and entire biomarker zones. We regarded TP as a good outcome patient whose zone was resected and TN as a poor outcome patient with incomplete resection. We classified FP and FN as a poor outcome patient whose zone was resected and a good outcome patient with incomplete resection, respectively. We computed the percentage of resected electrodes within a zone and considered it as resected if this percentage was above a specified *threshold*, using 50% as the most lenient condition^26^ to 75% as the most rigorous condition with 5% steps. We plotted the receiver operating characteristic (ROC) curve and computed the area under the curve (AUC) for each zone to assess their performance in outcome prediction.

We also utilized the logistic regression with leave one out cross validation method to examine if resection or non‐resection of a zone relates to outcome. We classified the test patient as TP if correctly predicted as good outcome, TN if correctly predicted as poor outcome, FP if incorrectly predicted as good outcome, and FN if incorrectly predicted as poor outcome. Thus, we estimated positive [PPV = TP/(TP + FP)] and negative predictive values [NPV = TN/(TN + FN)] for each zone.

### Statistical analysis

Statistical analyses were performed in MATLAB (The MathWorks, Inc.). We employed Wilcoxon rank‐sum test to compare distances of various zones from resection and their resection percentages between good and poor outcome patients. We applied Wilcoxon signed‐rank test to compare sensitivity, specificity, accuracy, and precision of zones in predicting resection in good outcome patients. This test was also used to compare distances of SOZ, propagation onset and spread, and entire biomarker zones from resection and their resection percentage in good outcome patients. We used Fisher's exact test to examine if the resection of each of the zones had an impact on outcome. We considered *p <* 0.05 significant and used the false discovery rate method^46^ to correct for multiple comparisons. We provide statistics as median (interquartile range) unless otherwise stated.

## Results

### Cohort

The cohort consists of 41 patients with a median age at surgery of 13 (6.75–16) years. Twenty‐five patients (61%) had seizure freedom at least 1 year after surgery. Nine patients (22%) had right hemisphere intracranial implantation, while 32 patients (78%) had left hemisphere. Seventeen patients (41%) had only subdural grid and strip contacts and 6 patients (15%) had only stereotactic contacts; 18 patients (44%) had both types of contacts implanted (Table 1). The time between the first seizure and surgery was shorter (*p =* 0.012) in good [4 (2–9) years] compared to poor outcome [7.5 (5–12.5) years] patients. There was no effect of gender, epilepsy localization, pathology, implantation side, inter‐contact distance, age at seizure onset, age at surgery, and years of follow‐up on outcome (Table 2).

**Table 2:** Patient's demographic information categorized by postsurgical outcome.

Characteristic	Total	Good outcome (Engel ≤1)	Poor outcome (Engel >1)	*p*
Patients, no.	41	25	16	–
Gender, no.				0.68^a^
Male	24	14	10	
Female	17	11	6	
Age at surgery, years, median (IQR)	13 (6.5–16)	11 (5.5–15.5)	13 (10–17.5)	0.13^b^
Age at seizure onset, years, median (IQR)	4 (1.4–7)	4 (1.25–8)	4 (1.75–7)	0.85^b^
Years between diagnosis and surgery, years, median (IQR)	5 (3–10)	4 (2–9)	7.5 (5–12.5)	0.01^b^ ^,^ *
Follow‐up, years, median (IQR).	1.42 (0.96–3.5)	1.42 (0.96–3.8)	1.42 (0.88–2.79)	0.42^b^
Implantation side, no.				0.71^a^
L	32	20	12	
R	9	5	4	
Inter‐contact Distance, mm, median (IQR).				
Grid and strips		9.2 (8.6–9.5)	9.3 (9.0–9.6)	0.99
SEEG		4.4 (3.5–4.9)	4.8 (4.1–5.0)	0.27
Resected contacts, no.	36 (28.8–41.3)	38 (31.8–52.5)	32 (25.5–38)	0.06^b^
Eplilepsy localization				0.53
Temporal		5	2	
Extra‐temporal		20	14	
Pathology				0.79^a^
NL	7	4	3	
MCD	28	18	10	
ACQ	6	3	3	

ACQ, acquired (i.e., stroke, neoplasm, and traumatic brain injury); IQR, interquartile range; L, left; MCD, malformation of cortical development (i.e., focal cortical dysplasia, polymicrogyria, gliosis, and tuberous sclerosis complex); NL, nonlesional; no., number; R, right.

^a^ Pearson chi‐squared test/Fisher's exact test.

^b^ Wilcoxon rank‐sum test.

* Significant comparisons with *p* ≤ 0.05.

### Spikes, ripples, and fast ripples propagations

Table 3 reports the total number of events and their propagation features. We detected spikes and ripples in all patients with a median rate of 2.3 (0.8–6.1) and 1.4 (0.8–2.3) events/min, respectively, and fast ripples in 12 (29%) patients with a rate of 0.2 (0–1.7) events/min. Fast ripples occurred at the lowest rate compared to spikes (*p <* 0.01) and ripples (*p =* 0.01). We detected a median of 46 (7–130) spike propagations at a rate of 7.0 (1.0–26.0) events/min. We observed spike propagations in 40 (98%) patients with a duration of 19 (15–24) ms, displacement of 8.3 (5.7–10.4) cm, and velocity of 4.8 (3.7–6.3) m/s. We identified a median of 25 (11–59) ripple propagations in 40 (98%) patients at a rate of 4.5 (1.9–12.0) events/min having a duration of 48 (41–46) ms, displacement of 7.6 (5.1–11.4) cm, and velocity of 1.9 (1.5–2.7) m/s. We found shorter duration (*p =* 0.03) of ripple propagations in good (compared to poor) outcome patients. We detected a median of 25 (12–69) fast ripple propagations in 7 out of 12 patients (58%) at a rate of 7.8 (2.5–21.3) events/min. Fast ripples showed a duration of 25 (13–28) ms, displacement of 3.8 (2.2–10.1) cm, and velocity of 2.8 (1.9–3.9) m/s.

**Table 3:** Features of individual events and propagations per outcome.

Individual events					*p*		
Feature	Outcome	Spike	Ripple	Fast ripple	S vs. R	S vs. FR	R vs. FR
No. of events	Good+Poor	730 (318–1843)	459 (262–721)	63 (1–514)	0.06	<0.01*	0.01*
	Good	564 (318–1772)	471 (262–829)	59 (1–466)	0.27	<0.01*	0.02*
	Poor	1033 (388–2281)	432 (274–670)	168 (43–1521)	0.13	0.32	0.4
	*p* ^†^	0.50	0.84	0.57			
Rate of events per channel [No./min]	Good+poor	2.3 (0.8–6.1)	1.4 (0.8–2.3)	0.2 (0–1.7)	0.10	<0.01*	0.02*
	Good	1.8 (0.9–5.9)	1.4 (0.8–2.3)	0.2 (0–1.6)	0.22	0.12	0.29
	Poor	2.6 (0.8–6.3)	1.3 (0.8–2.2)	0.6 (0.1–5.1)	0.30	0.44	0.42
	*p* ^†^	0.76	0.97	0.58			

Propagations					*p*		
Feature	Outcome	Spike	Ripple	Fast ripple	S vs. R	S vs. FR	R vs. FR
Rate of propagations [No./min]	Good+poor	7.0 (1.0–26.0)	4.5 (1.9–12.0)	7.8 (2.5–21.3)	0.70	0.80	0.48
	Good	3.8 (0.8–25.6)	5.0 (2.0–12.5)	7.8 (1.4–17.5)	0.94	1	0.89
	Poor	9.7 (1.9–25.5)	3.6 (1.9–11.1)	22.2 (5.2–39.2)	0.42	0.72	0.26
	*p* ^†^	0.49	0.77	0.57			
Percentage of propagating events [%]	Good+poor	18 (6–34)	24 (13–43)	51 (44–64)	0.13	0.01*	0.02*
	Good	18 (6–33)	25 (12–44)	49 (37–57)	0.83	0.66	0.17
	Poor	17 (9–36)	23 (14–40)	61 (54–68)	0.35	0.66	0.89
	*p* ^†^	0.85	0.97	0.38			
Duration [ms]	Good+poor	19 (15–24)	48 (41–46)	25 (13–28)	<0.001*	0.33	<0.01*
	Good	15 (15–25)	44 (34–57)	23 (9–25)	<0.001*	0.88	<0.01*
	Poor	20 (16–23)	56 (44–82)	36 (28–44)	<0.001*	0.06	0.16
	*p* ^†^	0.41	0.03*	0.09			
Displacement [cm]	Good+poor	8.3 (5.7–10.4)	7.6 (5.1–11.4)	3.8 (2.2–10.1)	0.68	0.33	0.24
	Good	7.8 (5.5–10.2)	6.9 (5.3–9.3)	3.8 (1.8–9.8)	0.94	0.27	0.26
	Poor	9.3 (8.0–13.2)	8.4 (4.5–12.1)	9.9 (2.6–17.2)	0.68	0.94	0.94
	*p* ^†^	0.11	0.73	0.57			
Velocity [m/s]	Good+poor	4.8 (3.7–6.3)	1.9 (1.5–2.7)	2.8 (1.9–3.9)	<0.001*	<0.01*	0.11*
	Good	4.5 (3.2–5.5)	2.2 (1.6–3.0)	2.7 (2.3–3.7)	<0.001*	0.05	0.17
	Poor	5.9 (4.4–6.6)	1.7 (1.2–2.0)	2.6 (1.0–4.2)	<0.001*	0.13	0.84
	*p* ^†^	0.13	0.13	1			
Latency variability [ms]	Good+poor	5 (5–8)	12 (8–19)	6 (5–8)	<0.001*	0.56	<0.01*
	Good	5 (4–8)	9 (7–16)	5 (4–6)	<0.001*	0.91	<0.01*
	Poor	5 (5–8)	15 (11–21)	9 (8–10)	<0.001*	0.18	0.07
	*p* ^†^	0.18	<0.02*	0.1			
Displacement variability [cm]	Good+poor	2.8 (1.8–3.8)	2.2 (1.6–3.6)	1.0 (0.9–2.0)	0.12	0.02	0.04
	Good	2.4 (1.8–3.1)	2.1 (1.4–3.0)	1.0 (0.8–2.0)	0.40	0.02	0.03
	Poor	3.7 (2.9–4.2)	2.9 (1.7–3.8)	2.3 (0.9–3.7)	0.07	0.44	0.64
	*p* ^†^	<0.01*	0.22	0.57			
Velocity variability [m/s]	Good+poor	4.6 (3.5–6.8)	2.3 (1.8–3.4)	3.8 (2.1–4.3)	<0.001*	0.05	0.17
	Good	4.3 (3.3–5.4)	2.5 (2.0–3.6)	3.8 (2.5–4.2)	<0.01*	0.24	0.34
	Poor	6.7 (4.8–7.5)	1.8 (1.4–2.5)	3.1 (1.5–4.7)	<0.001*	0.18	0.73
	*p* ^†^	0.02*	0.02*	1			
Number of channels in propagations	Good+poor	4 (3–5)	5 (4–5)	5 (4–6)	0.02	0.34	0.88
	Good	3 (3–6)	5 (4–6)	5 (4–5)	0.14	0.76	0.82
	Poor	4 (3–4)	4 (4–5)	5 (4–7)	0.05	0.44	1
	*p* ^†^	0.88	0.85	0.72			

FR, fast ripple; R, ripple; S, spike.

* Significant comparisons with *p* ≤ 0.05.

† *p*‐values for comparisons between *good versus poor* outcome patients.

Ripple propagations' duration was longer than spike and fast ripple propagations (*p <* 0.01). Spikes propagated the fastest followed by fast ripples (*p <* 0.001 for spike vs. ripple, and *p <* 0.01 for spike vs. fast ripple propagations); we found no differences in rate, displacement, and number of channels between spike, ripple, and fast ripple propagations (Table 3).

### Onset, spread, and SRO overlap

**Figure 2:**
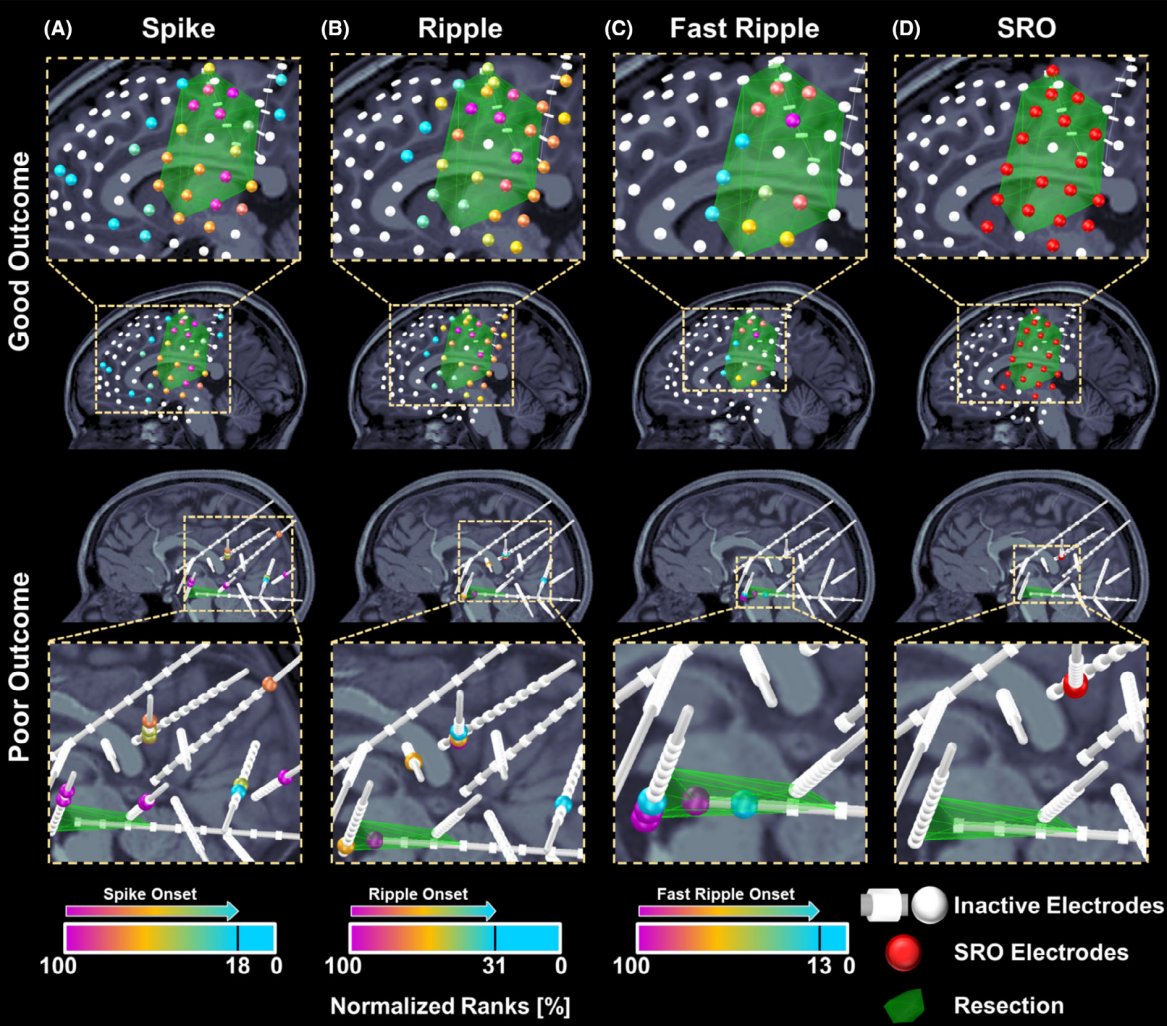
Electrodes ranking based on spike, ripple, and fast ripple propagations and spike–ripple onset overlap (SRO) zone in a good and a poor outcome patient. (A) Normalized ranks of electrodes contributing to spike propagations in a 9‐year‐old female patient (patient #8) with subdural and depth electrodes (good outcome) and a 10‐year‐old female patient (patient #27) with depth electrodes (poor outcome). Electrodes are depicted as color‐coded spheres; the color reflects normalized ranks from 100% (propagation onset) to 0% propagation end. Outlier electrodes or electrodes that did not contribute to propagations are depicted in white. (B) Electrodes' normalized ranks based on their participation in generating ripple propagations. (C) Electrode's normalized ranks based on fast ripple propagations. The optimal threshold to differentiate between onset and spread zones were calculated as 18.2%, 30.8%, and 13.0% for spike, ripple, and fast ripple propagations, respectively. (D) Electrodes present in both spike and ripple propagation onsets [i.e., the spike–ripple onset overlap zone (SRO)].

We mapped onset and spread zones for spike, ripple, and fast ripple propagations based on optimum thresholds obtained for each propagation type (18.2%, 30.8%, and 13.0% for spike, ripple, and fast ripple onsets, respectively). Figure 2 shows examples of onset, spread, and SRO and the extent they overlap with resection in a good and a poor outcome patient.

### Predicting resection in good outcome patients

**Figure 3:**
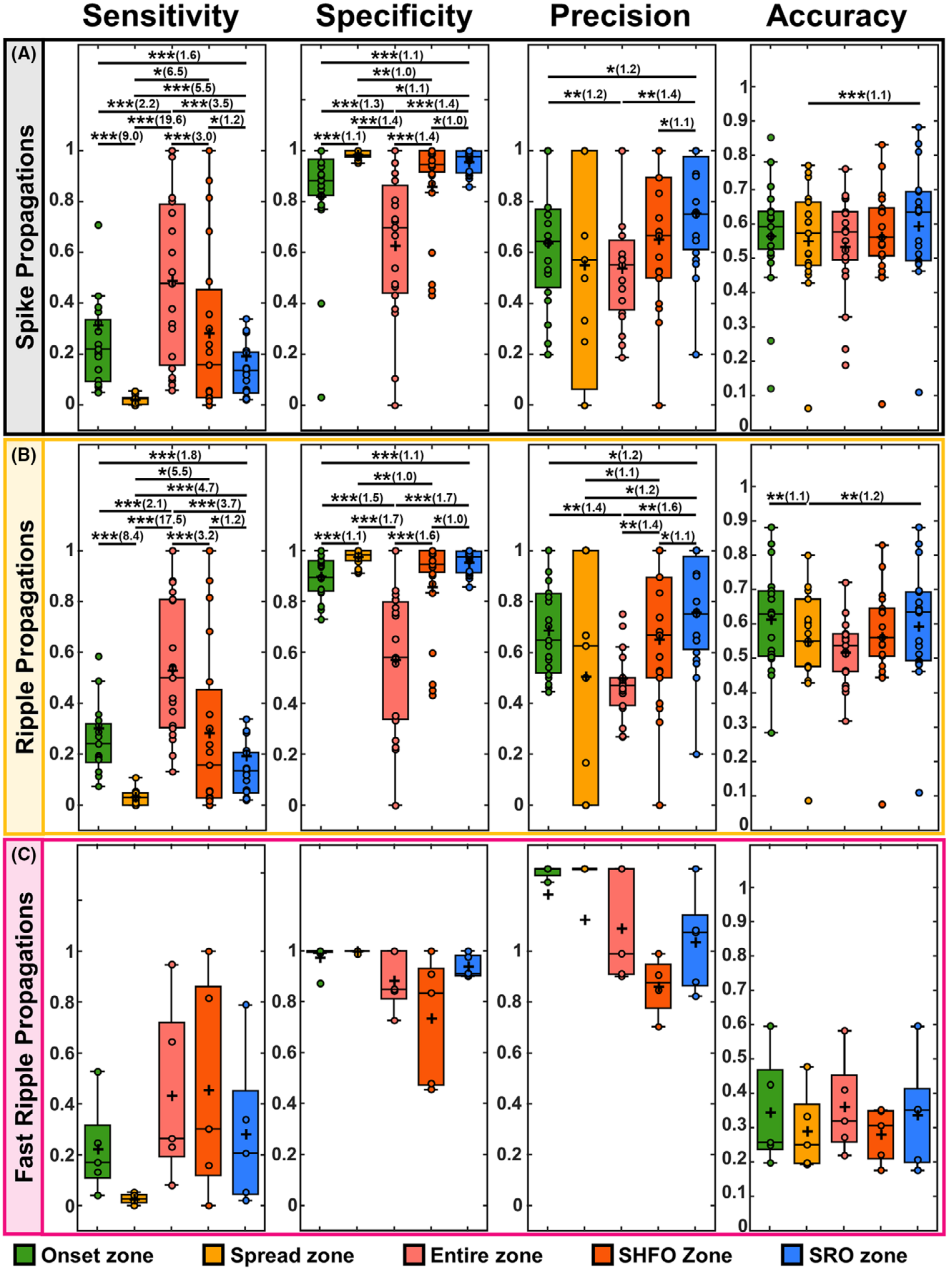
Statistics for prediction of resection in good outcome patients. (A) Sensitivity, specificity, precision, and accuracy of the onset and spread zone of spike propagations, the entire zone of spikes, the spikes co‐occurring with HFOs (SHFO) zone, and spike–ripple onset overlap zone (SRO) in predicting the resection zone of good outcome patients with SRO (21 patients). (B) Statistics for ripple onset and spread zones, the entire ripple zone, the SHFO, and the SRO in predicting resection zone of good outcome patients with SRO (21 patients). (C) Statistics for fast ripple onset and spread zones, the entire fast ripple zone, the SHFO, and the SRO in predicting resection zone of good outcome patients (5 patients). In the boxplots, the cross indicates the mean value, and the horizontal lines indicate the median value, lower and upper edges represent the 25th and 75th percentiles, whiskers extend to the 0th and 100th percentiles (excluding outliers) and points outside the whiskers represent the outliers (i.e., values that are at least 1.5 times the interquartile range below the 25th percentile or above the 75th percentile). The multiple comparisons issue was accounted for using the false discovery rate (FDR) correction. Pairs of significant differences are indicated by horizontal lines with asterisks above them: **p < 0.01, and ***p < 0.001. The effect size in significant comparisons is calculated as the ratio of the higher median value to the lower median value and is reported in parentheses after the asterisks.

We considered resection of good outcome patients as gold standard for estimating sensitivity, specificity, precision, and accuracy of different zones in predicting resection. Both spike and ripple onsets showed higher sensitivity in predicting resection, compared to spread zones and SRO (*p <* 0.001). SRO showed higher specificity in predicting resection compared to onset and zones of spikes and ripples (*p <* 0.001) and SHFO (*p <* 0.05). SRO was more precise in predicting resection compared to SHFO (*p <* 0.05), onsets (*p <* 0.01), ripples spread (*p <* 0.01), and zones (*p <* 0.01) of spikes and ripples. SRO (*p <* 0.001) was more accurate compared to spikes spread in predicting resection. Ripples onset and SRO were more accurate compared to spread (*p* < 0.01 for both comparisons) (Fig. 3A,B). We did not find any differences between fast ripples and SRO in predicting resection (Fig. 3C).

### Distance from resection and resection percentage of SOZ, onset, SHFO, and SRO

**Figure 4:**
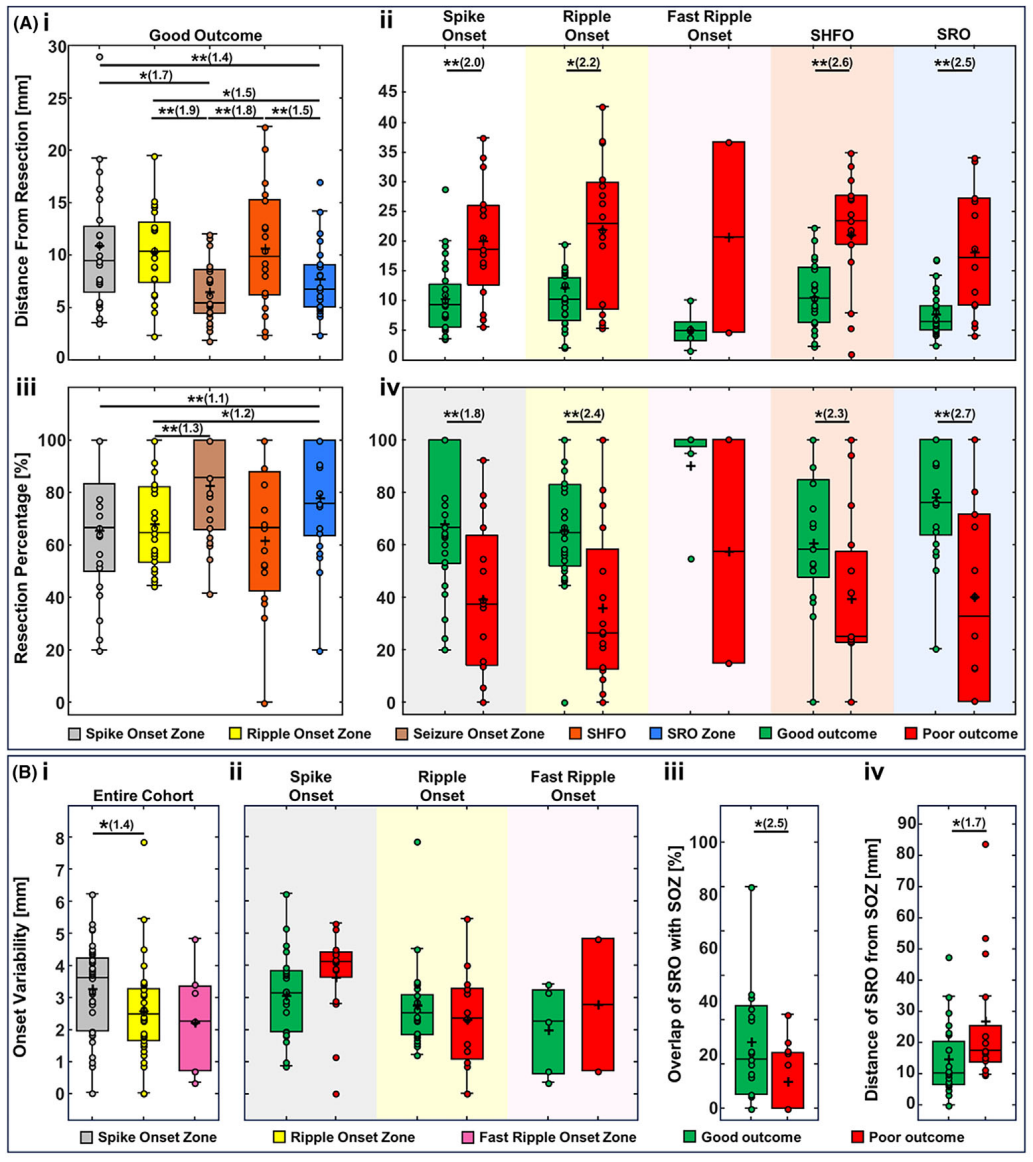
Distance from resection, resection percentage, and onset variability of different zones in predicting the epileptogenic zone. [A(i)] Comparing the distance of spike onset, ripple onset, seizure onset zone (SOZ), spike co‐occurring with HFOs (SHFO) zone, and spike–ripple onset overlap zone (SRO) from the resection zone in good outcome patients with SRO (21 patients). (ii) The distance from resection in good versus poor outcome patients for spike onset (good: 25 patients, poor: 15 patients), ripple onset (good: 24 patients, poor: 16 patients), fast ripple onset (good: 5 patients, poor: 2 patients), SHFO (good: 21 patients, poor: 15 patients), and SRO (good: 21 patients, poor: 14 patients). (iii) Comparing the overlap with resection for spike onset, ripple onset, SOZ, SHFO, and SRO in good outcome patients with SRO. (iv) The overlap with resection for spike onset, ripple onset, fast ripple onset, SHFO, and SRO in good versus poor outcome patients. [B(i)] comparing the onset variability of spike, ripple, and fast ripple onsets in good outcome patients. (ii) The onset variability of spike onset, ripple onset, SOZ and SRO in good versus poor outcome patients. (iii) Distance of SOZ from SRO and (iv) the percentage of overlap between SRO and SOZ in good versus poor outcome patients. In the boxplots, the cross indicates the mean value, and the horizontal lines indicate the median value, lower and upper edges represent the 25th and 75th percentiles, whiskers extend to the 0th and 100th percentiles (excluding outliers) and points outside the whiskers represent the outliers (i.e., values that are at least 1.5 times the interquartile range below the 25th percentile or above the 75th percentile). The multiple comparisons issue was accounted for using the false discovery rate (FDR) correction. Pairs of significant differences are indicated by horizontal lines with asterisks above them: *p < 0.05, **p < 0.01. The effect size in significant comparisons is calculated as the ratio of the higher median value to the lower median value and is reported in parentheses after the asterisks.

We provide statistics of distance from resection and resection percentage as mean ± standard deviation. In good outcome patients, SRO was closer to resection (7.7 ± 3.6 mm) compared to SHFO zone (10.7 ± 5.8 mm, *p =* 0.008), spike (10.9 ± 6.1 mm; *p =* 0.007), and ripple (10.4 ± 4.2 mm; *p =* 0.02) onset zones. SOZ was also closer to resection (6.5 ± 2.9 mm) compared to SHFO (*p =* 0.03), spike (*p =* 0.02), and ripple (*p =* 0.003) onset zones [Fig. 4A(i)]. The distance of SHFO and SRO as well as spike and ripple onsets were shorter in good (spikes: 10.7 ± 6.3 mm; ripples: 12.2 ± 10.4 mm; SHFO: 11.0 ± 5.7; SRO: 7.7 ± 3.6 mm) compared to poor outcome patients (spikes: 20.1 ± 10.0 mm, *p =* 0.003; ripples: 21.9 ± 12.1 mm, *p =* 0.013; SHFO: 21.4 ± 9.9 mm, *p =* 0.002; SRO: 18.1 ± 10.7 mm, *p =* 0.003). There was no difference in distance of fast ripple onset to resection for good versus poor outcome patients [Fig. 4A(ii)].

In good outcome patients, average resection was higher for SRO (79 ± 22%) compared to spike (66 ± 25%; *p =* 0.01) and ripple (68 ± 18%; *p =* 0.01) onsets. The average resection of SOZ (83 ± 19%) was also higher than ripples onset (68 ± 18%, *p =* 0.02). No differences were observed for resection between SHFO and other zones [Fig. 4A(iii)]. Resection of spike and ripple onsets as well as SHFO and SRO were higher in good (spikes: 68 ± 25%; ripples: 66 ± 23%; SHFO: 61 ± 29%; SRO: 78 ± 22%) compared to poor outcome patients (spikes: 39 ± 30%, *p =* 0.006; ripples: 36 ± 30%, *p =* 0.003; SHFO: 40 ± 32%, *p =* 0.04; SRO: 40 ± 38%, *p =* 0.005). Fast ripple onsets did not show differences between resection in good versus poor outcome patients [Fig. 4A(iv)]. We also studied distance from resection and resection percentages by considering only Engel I‐a as good outcome (Fig. S1). Also, considering the low fast ripples rate per channel (0.2 events/min, Table 3), we further investigated whether analysis of longer iEEG data will affect our findings: the analysis did not show differences in the distance of fast ripples zone from resection (Supplementary Material and Table S3). We also investigated the distance from resection and resection percentage of SRO using different cut‐off distances for defining resected electrodes (Supplementary Material on Defining Resected Electrodes and Fig. S2).

### Variability of spike, ripple, and fast ripple propagation onsets

Spike, ripple, and fast ripple propagations had a median onset variability of 3.6 (2.0–4.2), 2.5 (1.7–3.3), and 2.3 (0.7–3.4) mm, respectively [Fig. 4B(i)]. Τhe onset variability was higher in spike propagations compared to ripple propagations (*p =* 0.032). There was no difference of onset variability for spike [good: 3.1 (1.9–3.8) mm, poor: 4.1 (2.9–4.4) mm], ripple [good: 2.6 (1.9–3.2) mm, poor: 2.4 (1.1–3.3) mm], and fast ripple propagations [good: 2.3 (0.6–3.2), poor: 2.8 (0.7–4.8)] [Fig. 4B(ii)].

### Concordance of SRO and SOZ

Overlap between SRO and SOZ was higher in good [22.2 (6.2–46.3) %] compared to poor outcome patients [0 (0–25.0) %, *p =* 0.049] [Fig. 4B(iii)]. The distance of SRO from SOZ was lower in good [10.3 (6.5–22.7) mm] compared to poor outcome patients [17.5 (14.4–34.9) mm, *p =* 0.039] [Fig. 4B(iv)].

### Outcome prediction

**Figure 5:**
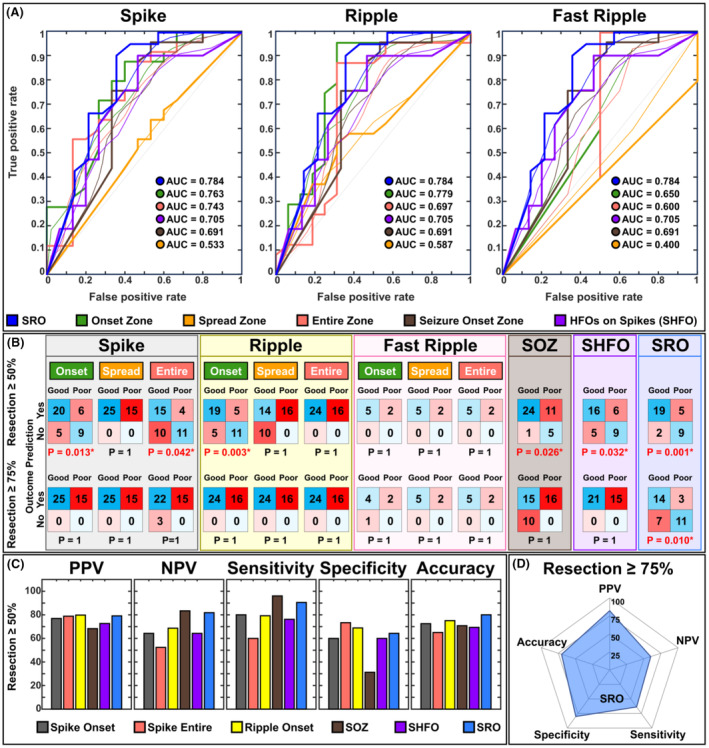
Outcome prediction results. (A) From left to right: receiver operating characteristic (ROC) curve and their area under the curve (AUC) for onset, spread, and entire zones of spike (40 patients), ripple (40 patients), and fast ripple propagations (7 patients), spike co‐occurring with HFOs (SHFO) zone (36 patients), the spike–ripple onset overlap zone (SRO, 35 patients), and the seizure onset zone (SOZ, 41 patients) as predictors of postsurgical outcome. (B) Confusion matrices for each of the zones in predicting the surgical outcome at resection threshold of 50% and resection threshold of 75% using the logistic regression with leave one out cross validation method. (C) from left to right: positive predictive value (PPV), negative predictive value (NPV), sensitivity, specificity, and accuracy of each of the zones for predicting the outcome corresponding to the resection threshold of 50%. (D) Spider plot of PPV, NPV, sensitivity, specificity, and accuracy of each of the zones for predicting the outcome corresponding to the resection threshold of 75%.

The AUC of SRO for predicting outcome was higher than the AUC of the SOZ, zones of spikes and ripples, their onsets and spreads, and fast ripple zones and spreads (Fig. 5A). By considering resection threshold of 50%, we observed that the resection of onsets of spikes and ripples, the spikes zone, SOZ, SHFO, and SRO predicted outcome (Fig. 5B,C). By applying stricter criteria (threshold 75%), only resection of SRO predicted outcome (*p =* 0.01) (Fig. 5B–D and Table S2). We also evaluated the outcome predictability of different zones by considering only Engel I‐a as good outcome (Fig. S3).

## Discussion

By mapping the spatiotemporal propagations of different interictal biomarkers, we show that SRO is a highly specific and precise predictor of the EZ and surgical outcome in patients with DRE. SRO presents higher predictive performance compared to fast ripples and is more advantageous because it can be seen in most patients (SRO in 85% vs. fast ripples in 29%). Moreover, it holds higher outcome predictability when stricter resection criteria are considered compared to previous studies.^9^, ^26^ This notion derives from our main findings: (i) SRO is closer to resection compared to SHFO and individual onsets of spike and ripple propagations in good outcome patients; (ii) SRO is closer to resection in good versus poor outcome patients; (iii) resection of SRO is higher compared to resection of individual onsets of spike and ripple propagations in good outcome patients; (iv) SRO has higher resection in good versus poor outcome patients; (v) SRO predicts resection of good outcome patients with higher specificity and precision compared to individual onsets of spike and ripple propagations; (vi) SRO predicts resection of good outcome patients with higher accuracy compared to areas of spread for spike and ripple propagations; (vii) onset zones of spike and ripple propagations as well as SOZ, SHFO and SRO predict outcome; (viii) when stricter criteria are used for defining resection, the spike and ripple onsets as well as SOZ and SHFO lose outcome predictability while only SRO remains to predict outcome; and (ix) resection of spread zones and zones of spikes and ripples do not predict outcome.

### SRO delineates the EZ and predicts surgical outcome

Previous studies examined the propagation of interictal biomarkers, such as spikes^9^, ^25^ or ripples.^32^, ^47^ Studies on spikes found that iEEG electrodes with earlier peaks are more epileptogenic (compared to spread areas) and related to the EZ.^25^, ^29^ By using a similar strategy, other studies related iEEG electrodes with higher early ripples rates to the EZ.^26^ Yet, results of this body of literature are contradictory and inconclusive.^10^, ^32^ Studies from our group with iEEG^26^ as well as noninvasive electrophysiological methods^6^ have shown that ripple propagations onset is predictive of the EZ and outcome. Our study confirms these findings using the same parameters defining resection (percentage >50%). Yet, when stricter criteria (percentage >70%) are used, these findings do not hold significance (Fig. 5B,C and Table S2). While the SOZ and individual spike or ripple propagation onsets pointed to the EZ, their near‐complete resection (percentage >70%) did not predict outcome (Fig. 5B,C and Table S2). This may indicate that individual onsets either miss parts of the EZ or include excessive resection areas, whereas SRO predicts the EZ with the highest specificity and precision as it focuses on common onset generators of spike and ripple propagations, thereby predicting outcome. We observed a higher degree of concordance between SRO and SOZ in good outcome patients; the two zones were closer to each other and had a higher overlap [Fig. 4B(iii,iv)]. However, this overlap was marginal (~20%) even in good outcome patients. This marginal overlap may arise from the limited iEEG coverage.

Fast ripples originate from fixed and focal pathologically interconnected neuronal clusters,^23^ which form networks.^48^ These fast ripples are reported to prime spike propagations,^22^ yet, removing fast ripple areas preceding spikes does not guarantee seizure freedom.^22^ Fast ripples also propagate^47^, ^49^; and networks of propagating fast ripples may increase the likelihood of triggering epileptiform spikes. However, these networks resection is not related to outcome.^49^ Here, we found no strong association between outcome and the SHFO zone. We also found no association between resection of different fast ripple zones and outcome.

### Features of spikes, ripples, and fast ripples spatiotemporal propagations

Several studies have examined spatiotemporal characteristics of propagating interictal biomarkers.^9^, ^24^, ^25^, ^26^, ^32^, ^49^ However, a gap exists in the literature for studies that comprehensively examine and utilize these features within the same cohort. Here, we quantify several spatiotemporal features for spikes, ripples, and fast ripples, and examine the clinical significance of combining these features into a single biomarker.

Previous studies on spike propagations report a range of velocities; at the cellular (or source space) level, spikes propagate with a velocity of ~0.1 up to 0.5 m/s,^9^, ^50^ while at the sensor level, higher propagation values are reported (≥2 m/s).^26^, ^51^, ^52^ Here, we observed a velocity of ~5 m/s in line with these previous findings. We found that spikes propagate ~2.5 times faster than ripples^26^ (median velocity of 1.9 m/s) and ~ 1.7 times faster than fast ripples (median velocity of 2.8 m/s) (Table 3). Studies on spike and fast ripple generation mechanisms have shown that these two biomarkers are caused by increased excitability both at the pyramidal cells level and at broader network levels, with different pathological discharge patterns at the pyramidal cells triggering either spikes or fast ripples.^53^ Fast ripples precede and prime spikes that tend to propagate.^22^, ^49^ In accordance with these studies, we observed here no differences for duration and latency, displacement, and velocity variability between spike and fast ripple propagations. Contrarily, ripple propagations lasted longer than spike and fast ripple propagations and had greater latency and velocity variability compared to spike propagations. Spikes are known to arise from a brief imbalance between excitatory (glutamatergic) and inhibitory (GABAergic) neurotransmission,^54^ leading to a sudden, synchronous discharge of a group of neurons; similar to fast ripples that seem to occur during transitory hyper synchronization of neuronal activity.^55^ Ripples on the other hand originate from synchronous firing of pyramidal cells, such as in slow‐wave sleep or quiet wakefulness, followed by suppression of inhibitory interneurons. These differences in underlying mechanisms of spike, ripple, and fast ripple propagations may explain the observed differences in their propagation features.

Spike, ripple, and fast ripple propagation onsets showed low onset variability of ~4 mm; this indicates a probable uniform and interconnected epileptogenic network at the source level. Spike propagations showed larger onset variability compared to ripple propagations [Fig. 4B(i)]. Spikes may propagate across lobes or hemispheres via association and commissural fibers, which cover short and long paths.^28^, ^52^ Studies have reported fast ripples and their propagation to be more spatially constrained.^32^, ^56^ Ripples were found to propagate in larger networks compared to fast ripples.^32^ Here, we found propagation displacement of ~8 cm for spikes and ripples, and ~ 4 cm for fast ripples. Such a displacement is physiologically relevant considering fibers' lengths reaching up to ~16 cm.^57^ It should be noted that these displacements were obtained from Euclidian distances between electrodes, which may not represent the actual length of anatomical propagation pathways.

Both seizures and interictal spike propagations are more spatially organized in good outcome patients,^24^, ^58^ implying that spike generating regions are clustered together in seizure‐free patients. Here, we found that spike propagations have a lower displacement variability, in good (compared to poor) outcome patients (Table 3). Our findings may suggest a more focal and highly interconnected EZ in seizure‐free patients. Moreover, we found shorter ripple propagation duration and latency variability of ripple propagations in good outcome patients, which is in accordance with the presumption of higher inter‐connectivity and recruitment of surrounding neurons in the epileptogenic tissue in seizure‐free patients.^24^ The current study contributes to the existing literature by offering a comprehensive analysis of spatiotemporal features of interictal biomarkers in a large cohort of 41 patients with DRE.

### Limitations

We examined a heterogeneous single‐center cohort encompassing various pathologies. Larger scale, multicenter studies are warranted to facilitate patient stratification into more homogenous subgroups. Moreover, iEEG has limited spatial resolution and coverage, which can lead to oversighting critical epileptogenic sites. Future investigations examining spike and HFO propagations at source level may mitigate challenges related to iEEG inherent limitations. Due to iEEG spatial resolution constraints, we were unable to investigate propagation throughout brain entirety. Future studies may leverage noninvasive neuroimaging techniques having full coverage. Electrode implantations consisted of stereotactic contacts and grid/strip electrodes or combinations of both. Yet, grid/strip electrodes measure cortical surface activity while stereotactic contacts measure activity across distant cortical structures traversing white matter. Future studies should examine whether current results hold for both electrode types.

## Conclusion

Spike and ripple propagations, originating from the same generator (SRO), are precise predictors of the EZ and surgical outcome in most patients with DRE. The SRO's specificity and precision are comparable to fast ripples, but SRO is more advantageous because it can be seen in most patients with DRE. Our findings highlight the potential clinical utility of SRO as an EZ biomarker in children with DRE. Prospective studies are required to confirm these findings and fully establish the effectiveness of SRO in improving surgical outcome.

## Author Contributions

S.J. and C.P. contributed to the conception and design of the study. S.J., M.A.G.M., L.F., and C.P. validated the methodology and analyzed the data. S.J., M.A.G.M., E.T., J.R.M., J.B., P.L.P, and C.P. acquired the data. S.J. and M.A.G.M., and C.P. contributed to drafting a significant portion of the original manuscript and figures. S.J, M.A.G.M., E.T., M.S.P., J.R.M., J.B., S.S.D.S., P.L.P, and C.P. reviewed and edited the manuscript. C.P. supervised the study. C.P. provided resources.

## Conflict of Interest

The authors have no conflict of interest to report.

## Supporting information


Figure S1.

Figure S2.

Figure S3.

Table S1.

Table S2.

Table S3.

Data S1.

## Data Availability

The data are available from the corresponding author upon request.

## References

[1] Englot DJ , Chang EF . Rates and predictors of seizure freedom in resective epilepsy surgery: an update. Neurosurg Rev. 2014;37(3):389‐404; discussion 404–405. 24497269 10.1007/s10143-014-0527-9 PMC5257205

[2] Papadelis C , Perry MS . Localizing the epileptogenic zone with novel biomarkers. Semin Pediatr Neurol. 2021;39:100919. 34620466 10.1016/j.spen.2021.100919 PMC8501232

[3] Rijal S , Corona L , Perry MS , et al. Functional connectivity discriminates epileptogenic states and predicts surgical outcome in children with drug resistant epilepsy. Sci Rep. 2023;13(1):9622. 37316544 10.1038/s41598-023-36551-0 PMC10267141

[4] Papadelis C , Tamilia E , Stufflebeam S , et al. Interictal high frequency oscillations detected with simultaneous magnetoencephalography and electroencephalography as biomarker of pediatric epilepsy. J Vis Exp. 2016;(118):54883. 28060325 10.3791/54883 PMC5226354

[5] Fujiwara H , Kadis DS , Greiner HM , et al. Clinical validation of magnetoencephalography network analysis for presurgical epilepsy evaluation. Clin Neurophysiol. 2022;142:199‐208. 36063669 10.1016/j.clinph.2022.07.506

[6] Tamilia E , Matarrese MAG , Ntolkeras G , et al. Noninvasive mapping of ripple onset predicts outcome in epilepsy surgery. Ann Neurol. 2021;89(5):911‐925. 33710676 10.1002/ana.26066 PMC8229023

[7] Akiyama T , McCoy B , Go CY , et al. Focal resection of fast ripples on extraoperative intracranial EEG improves seizure outcome in pediatric epilepsy. Epilepsia. 2011;52(10):1802‐1811. 21801168 10.1111/j.1528-1167.2011.03199.x

[8] Corona L , Tamilia E , Perry MS , et al. Non‐invasive mapping of epileptogenic networks predicts surgical outcome. Brain. 2023;146(5):1916‐1931. 36789500 10.1093/brain/awac477 PMC10151194

[9] Matarrese MAG , Loppini A , Fabbri L , et al. Spike propagation mapping reveals effective connectivity and predicts surgical outcome in epilepsy. Brain. 2023;146:3898‐3912. 37018068 10.1093/brain/awad118 PMC10473571

[10] Azeem A , von Ellenrieder N , Hall J , Dubeau F , Frauscher B , Gotman J . Interictal spike networks predict surgical outcome in patients with drug‐resistant focal epilepsy. Ann Clin Transl Neurol. 2021;8(6):1212‐1223. 33951322 10.1002/acn3.51337 PMC8164864

[11] Frauscher B , Bartolomei F , Kobayashi K , et al. High‐frequency oscillations: the state of clinical research. Epilepsia. 2017;58(8):1316‐1329. 28666056 10.1111/epi.13829 PMC5806699

[12] Jacobs J , Zijlmans M , Zelmann R , et al. High‐frequency electroencephalographic oscillations correlate with outcome of epilepsy surgery. Ann Neurol. 2010;67(2):209‐220. 20225281 10.1002/ana.21847 PMC3769290

[13] Schönberger J , Birk N , Lachner‐Piza D , Dümpelmann M , Schulze‐Bonhage A , Jacobs J . High‐frequency oscillations mirror severity of human temporal lobe seizures. Ann Clin Transl Neurol. 2019;6(12):2479‐2488. 31750633 10.1002/acn3.50941 PMC6917313

[14] Hussain SA , Mathern GW , Hung P , Weng J , Sankar R , Wu JY . Intraoperative fast ripples independently predict postsurgical epilepsy outcome: comparison with other electrocorticographic phenomena. Epilepsy Res. 2017;135:79‐86. 28644979 10.1016/j.eplepsyres.2017.06.010 PMC5568451

[15] Tobochnik S , Bateman LM , Akman CI , et al. Tracking multisite seizure propagation using ictal high‐gamma activity. J Clin Neurophysiol. 2022;39(7):592‐601. 34812578 10.1097/WNP.0000000000000833 PMC8611231

[16] Cho JR , Koo DL , Joo EY , et al. Resection of individually identified high‐rate high‐frequency oscillations region is associated with favorable outcome in neocortical epilepsy. Epilepsia. 2014;55(11):1872‐1883. 25266626 10.1111/epi.12808

[17] Zijlmans M , Jacobs J , Kahn YU , Zelmann R , Dubeau F , Gotman J . Ictal and interictal high frequency oscillations in patients with focal epilepsy. Clin Neurophysiol. 2011;122(4):664‐671. 21030302 10.1016/j.clinph.2010.09.021 PMC3771929

[18] Wang S , Wang IZ , Bulacio JC , et al. Ripple classification helps to localize the seizure‐onset zone in neocortical epilepsy. Epilepsia. 2013;54(2):370‐376. 23106394 10.1111/j.1528-1167.2012.03721.x

[19] Schönberger J , Knopf A , Klotz KA , Dümpelmann M , Schulze‐Bonhage A , Jacobs J . Distinction of physiologic and epileptic ripples: an electrical stimulation study. Brain Sci. 2021;11(5):538. 33923317 10.3390/brainsci11050538 PMC8146715

[20] Roehri N , Pizzo F , Lagarde S , et al. High‐frequency oscillations are not better biomarkers of epileptogenic tissues than spikes. Ann Neurol. 2018;83(1):84‐97. 29244226 10.1002/ana.25124

[21] Bernardo D , Nariai H , Hussain SA , et al. Visual and semi‐automatic non‐invasive detection of interictal fast ripples: a potential biomarker of epilepsy in children with tuberous sclerosis complex. Clin Neurophysiol. 2018;129(7):1458‐1466. 29673547 10.1016/j.clinph.2018.03.010

[22] Weiss SA , Fried I , Jerome EJ , et al. Fast ripples reflect increased excitability that primes epileptiform spikes. Brain Communications. 2023;5(5):fcad242. 37869578 10.1093/braincomms/fcad242 PMC10587774

[23] Bragin A , Wilson CL , Engel J . Spatial stability over time of brain areas generating fast ripples in the epileptic rat. Epilepsia. 2003;44(9):1233‐1237. 12919396 10.1046/j.1528-1157.2003.18503.x

[24] Tomlinson SB , Bermudez C , Conley C , Brown MW , Porter BE , Marsh ED . Spatiotemporal mapping of Interictal spike propagation: a novel methodology applied to pediatric intracranial EEG recordings. Front Neurol. 2016;7:229. 28066315 10.3389/fneur.2016.00229 PMC5165024

[25] Mălîia MD , Meritam P , Scherg M , et al. Epileptiform discharge propagation: analyzing spikes from the onset to the peak. Clin Neurophysiol. 2016;127(4):2127‐2133. 26818882 10.1016/j.clinph.2015.12.021

[26] Tamilia E , Park E‐H , Percivati S , et al. Surgical resection of ripple onset predicts outcome in pediatric epilepsy. Ann Neurol. 2018;84(3):331‐346. 30022519 10.1002/ana.25295

[27] Alarcon G , Garcia Seoane JJ , Binnie CD , et al. Origin and propagation of interictal discharges in the acute electrocorticogram. Implications for pathophysiology and surgical treatment of temporal lobe epilepsy. Brain. 1997;120(Pt 12):2259‐2282. 9448581 10.1093/brain/120.12.2259

[28] Emerson RG , Turner CA , Pedley TA , Walczak TS , Forgione M . Propagation patterns of temporal spikes. Electroencephalogr Clin Neurophysiol. 1995;94(5):338‐348. 7774520 10.1016/0013-4694(94)00316-d

[29] Diamond JM , Withers CP , Chapeton JI , Rahman S , Inati SK , Zaghloul KA . Interictal discharges in the human brain are travelling waves arising from an epileptogenic source. Brain. 2023;146(5):1903‐1915. 36729683 10.1093/brain/awad015 PMC10411927

[30] Maharathi B , Wlodarski R , Bagla S , et al. Interictal spike connectivity in human epileptic neocortex. Clin Neurophysiol. 2019;130(2):270‐279. 30605889 10.1016/j.clinph.2018.11.025 PMC8543819

[31] Smith EH , Liou J , Merricks EM , et al. Human interictal epileptiform discharges are bidirectional traveling waves echoing ictal discharges. elife. 2022;11:e73541. 35050851 10.7554/eLife.73541 PMC8813051

[32] González Otárula KA , von Ellenrieder N , Cuello‐Oderiz C , Dubeau F , Gotman J . High‐frequency oscillation networks and surgical outcome in adult focal epilepsy. Ann Neurol. 2019;85(4):485‐494. 30786048 10.1002/ana.25442

[33] Matarrese MAG , Loppini A , Jahromi S , et al. Electric source imaging on intracranial EEG localizes spatiotemporal propagation of Interictal spikes in children with epilepsy. Annu Int Conf IEEE Eng Med Biol Soc. 2021;2021:2668‐2671. 34891801 10.1109/EMBC46164.2021.9630246 PMC8928574

[34] Olejarczyk E , Gotman J , Frauscher B . Region‐specific complexity of the intracranial EEG in the sleeping human brain. Sci Rep. 2022;12(1):451. 35013431 10.1038/s41598-021-04213-8 PMC8748934

[35] Frauscher B , von Ellenrieder N , Ferrari‐Marinho T , Avoli M , Dubeau F , Gotman J . Facilitation of epileptic activity during sleep is mediated by high amplitude slow waves. Brain. 2015;138(Pt 6):1629‐1641. 25792528 10.1093/brain/awv073 PMC4614129

[36] Sammaritano M , Gigli GL , Gotman J . Interictal spiking during wakefulness and sleep and the localization of foci in temporal lobe epilepsy. Neurology. 1991;41(2_part_1):290. 1992379 10.1212/wnl.41.2_part_1.290

[37] Iandolo G , Chourasia N , Ntolkeras G , et al. Changes in the functional brain network of children undergoing repeated epilepsy surgery: an EEG source connectivity study. Diagnostics (Basel). 2021;11(7):123. 34359317 10.3390/diagnostics11071234 PMC8306224

[38] Ono M , Kubik S , Abernathey CD . Atlas of the Cerebral Sulci. G. Thieme Verlag; 1990. https://books.google.com/books?id=xroe986wtkEC

[39] Kim D , Joo EY , Seo D‐W , et al. Accuracy of MEG in localizing irritative zone and seizure onset zone: quantitative comparison between MEG and intracranial EEG. Epilepsy Res. 2016;127:291‐301. 27693985 10.1016/j.eplepsyres.2016.08.013

[40] Engel J Jr . Surgery for seizures. N Engl J Med. 1996;334(10):647‐653. 8592530 10.1056/NEJM199603073341008

[41] Scheuer ML , Wilson SB , Antony A , Ghearing G , Urban A , Bagić AI . Seizure detection: Interreader agreement and detection algorithm assessments using a large dataset. J Clin Neurophysiol. 2021;38(5):439‐447. 32472781 10.1097/WNP.0000000000000709 PMC8404956

[42] Navarrete M , Alvarado‐Rojas C , Le Van QM , Valderrama M . RIPPLELAB: a comprehensive application for the detection, analysis and classification of high frequency oscillations in electroencephalographic signals. PLoS One. 2016;11(6):e0158276. 27341033 10.1371/journal.pone.0158276 PMC4920418

[43] Gerstl JVE , Kiseleva A , Imbach L , Sarnthein J , Fedele T . High frequency oscillations in relation to interictal spikes in predicting postsurgical seizure freedom. Sci Rep. 2023;13(1):21313. 38042925 10.1038/s41598-023-48764-4 PMC10693609

[44] Conrad EC , Revell AY , Greenblatt AS , et al. Spike patterns surrounding sleep and seizures localize the seizure‐onset zone in focal epilepsy. Epilepsia. 2023;64(3):754‐768. 36484572 10.1111/epi.17482 PMC10045742

[45] Shamas M , Yeh HJ , Fried I , Engel J Jr , Staba RJ . High‐rate leading spikes in propagating spike sequences predict seizure outcome in surgical patients with temporal lobe epilepsy. Brain Commun. 2023;5(6):fcad289. 37953846 10.1093/braincomms/fcad289 PMC10636565

[46] Benjamini Y , Drai D , Elmer G , Kafkafi N , Golani I . Controlling the false discovery rate in behavior genetics research. Behav Brain Res. 2001;125(1–2):279‐284. 11682119 10.1016/s0166-4328(01)00297-2

[47] Jahromi S , Matarrese MAG , Tamilia E , et al. Mapping propagation of Interictal spikes, ripples, and fast ripples in intracranial EEG of children with refractory epilepsy. Annu Int Conf IEEE Eng Med Biol Soc. 2021;2021:194‐197. 34891270 10.1109/EMBC46164.2021.9630250 PMC8896264

[48] Bragin A , Wilson CL , Engel JJ . Chronic epileptogenesis requires development of a network of pathologically interconnected neuron clusters: a hypothesis. Epilepsia. 2000;41(Suppl 6):S144‐S152. 10999536 10.1111/j.1528-1157.2000.tb01573.x

[49] Weiss SA , Sheybani L , Seenarine N , et al. Delta oscillation coupled propagating fast ripples precede epileptiform discharges in patients with focal epilepsy. Neurobiol Dis. 2022;175:105928. 36403895 10.1016/j.nbd.2022.105928 PMC12323987

[50] Zhang M , Ladas TP , Qiu C , Shivacharan RS , Gonzalez‐Reyes LE , Durand DM . Propagation of epileptiform activity can be independent of synaptic transmission, gap junctions, or diffusion and is consistent with electrical field transmission. J Neurosci. 2014;34(4):1409‐1419. 24453330 10.1523/JNEUROSCI.3877-13.2014 PMC3898297

[51] Alarcon G , Guy CN , Binnie CD , Walker SR , Elwes RD , Polkey CE . Intracerebral propagation of interictal activity in partial epilepsy: implications for source localisation. J Neurol Neurosurg Psychiatry. 1994;57(4):435‐449. 8163992 10.1136/jnnp.57.4.435 PMC1072872

[52] Baumgartner C , Lindinger G , Ebner A , et al. Propagation of interictal epileptic activity in temporal lobe epilepsy. Neurology. 1995;45(1):118‐122. 7824100 10.1212/wnl.45.1.118

[53] Demont‐Guignard S , Benquet P , Gerber U , Biraben A , Martin B , Wendling F . Distinct hyperexcitability mechanisms underlie fast ripples and epileptic spikes. Ann Neurol. 2012;71(3):342‐352. 22451202 10.1002/ana.22610

[54] Avoli M , de Curtis M . GABAergic synchronization in the limbic system and its role in the generation of epileptiform activity. Prog Neurobiol. 2011;95(2):104‐132. 21802488 10.1016/j.pneurobio.2011.07.003 PMC4878907

[55] Bragin A , Mody I , Wilson CL , Engel JJ . Local generation of fast ripples in epileptic brain. J Neurosci. 2002;22(5):2012‐2021. 11880532 10.1523/JNEUROSCI.22-05-02012.2002 PMC6758883

[56] Staba RJ , Wilson CL , Bragin A , Fried I , Engel J Jr . Quantitative analysis of high‐frequency oscillations (80‐500 Hz) recorded in human epileptic hippocampus and entorhinal cortex. J Neurophysiol. 2002;88(4):1743‐1752. 12364503 10.1152/jn.2002.88.4.1743

[57] Bajada CJ , Schreiber J , Caspers S . Fiber length profiling: a novel approach to structural brain organization. NeuroImage. 2019;186:164‐173. 30399419 10.1016/j.neuroimage.2018.10.070

[58] Martinet L‐E , Ahmed OJ , Lepage KQ , Cash SS , Kramer MA . Slow spatial recruitment of neocortex during secondarily generalized seizures and its relation to surgical outcome. J Neurosci. 2015;35(25):9477‐9490. 26109670 10.1523/JNEUROSCI.0049-15.2015 PMC4478258

