# Supplementary material for: Overlap of spike and ripple propagation onset predicts surgical outcome in epilepsy
Source: Ann Clin Transl Neurol. 2024 Oct 7;11(10):2530–47. doi: 10.1002/acn3.52156 (PMC11514932; doi:10.1002/acn3.52156)
Supplement: Supplementary file 1 — Figure S1. [file ACN3-11-2530-s002.docx]

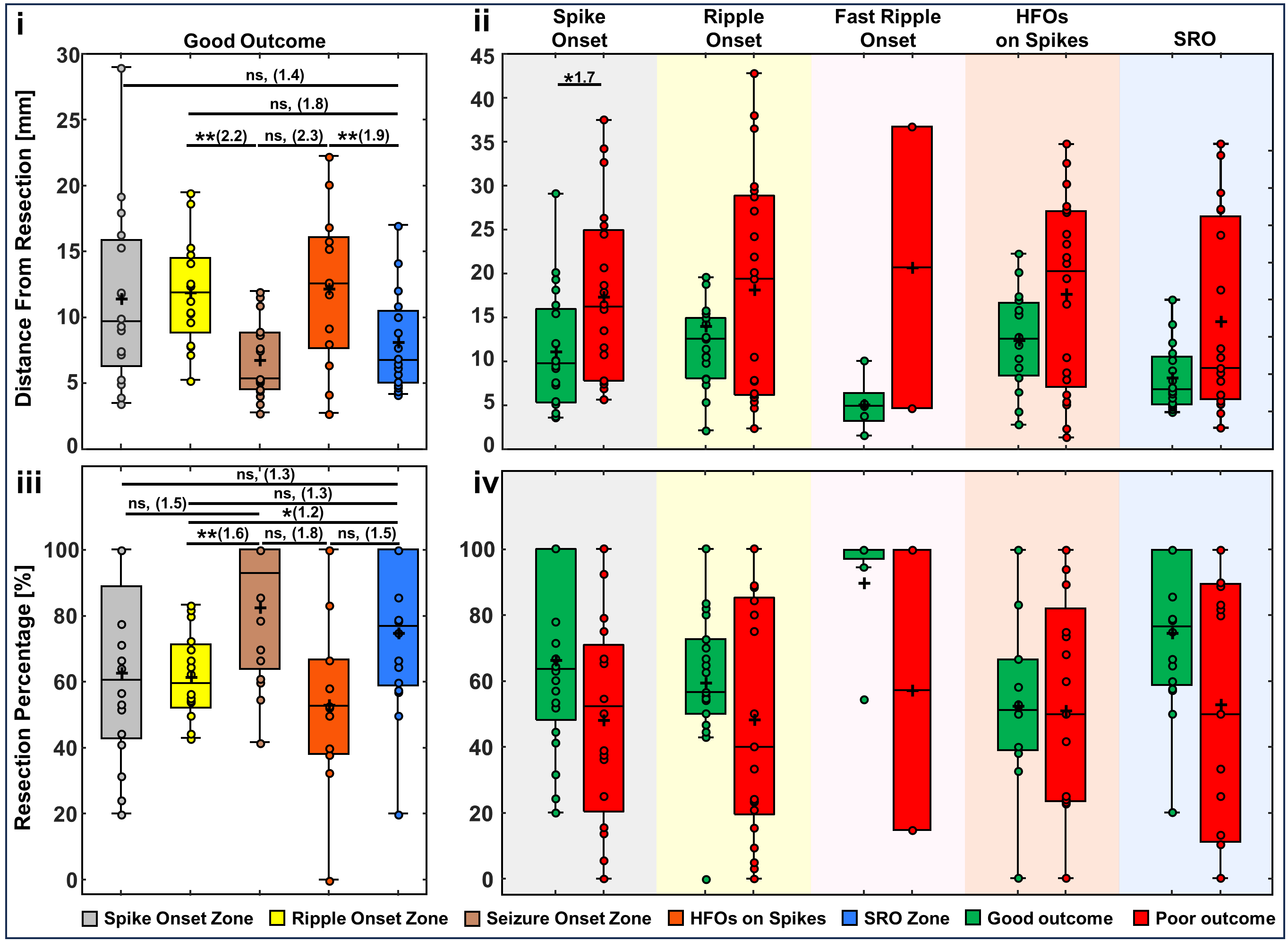


**Supplementary Figure S1.** **Spikes, ripples, fast ripples, and different zones predicting the epileptogenic zone, considering Engel I-a as good outcome. (i)** Comparing the distance of spike onset, ripple onset, seizure onset zone (SOZ), spike co-occurring with HFOs (SHFO) zone, and spike-ripple onset overlap zone (SRO) from the resection zone in good outcome patients (16 patients). **(ii)** the distance from resection of spike onset (good: 20 patients, poor: 20 patients), ripple onset (good: 19 patients, poor: 21 patients), fast ripple onset (good: 5 patients, poor: 2 patients), SHFO (good: 16 patients, poor: 20 patients), and SRO (good: 16 patients, poor: 19 patients) in good vs. poor outcome patients. **(iii)** comparing the overlap with resection for spike onset, ripple onset, SOZ, SHFO, and SRO in good outcome patients. **(iv)** the overlap with resection for spike onset, ripple onset, fast ripple onset, SHFO, and SRO in good vs. poor outcome patients. In the boxplots, the cross indicates the mean value, and the horizontal lines indicate the median value, lower and upper edges represent the 25th and 75th percentiles, whiskers extend to the 0th and 100th percentiles (excluding outliers) and points outside the whiskers represent the outliers (i.e., values that are at least 1.5 times the interquartile range below the 25th percentile or above the 75th percentile). The multiple comparisons issue was accounted for using the false discovery rate (FDR) correction. Pairs of significant differences are indicated by horizontal lines with asterisks above them: **p<*0.05, ***p<*0.01. The effect size in significant comparisons is calculated as the ratio of the higher median value to the lower median value and is reported in parentheses after the asterisks. Comparisons that had a significant p-value prior to applying the multiple comparisons correction are shown as not significant (ns), with their effect size reported.
