# Supplementary material for: Overlap of spike and ripple propagation onset predicts surgical outcome in epilepsy
Source: Ann Clin Transl Neurol. 2024 Oct 7;11(10):2530–47. doi: 10.1002/acn3.52156 (PMC11514932; doi:10.1002/acn3.52156)
Supplement: Supplementary file 2 — Figure S2. [file ACN3-11-2530-s005.docx]

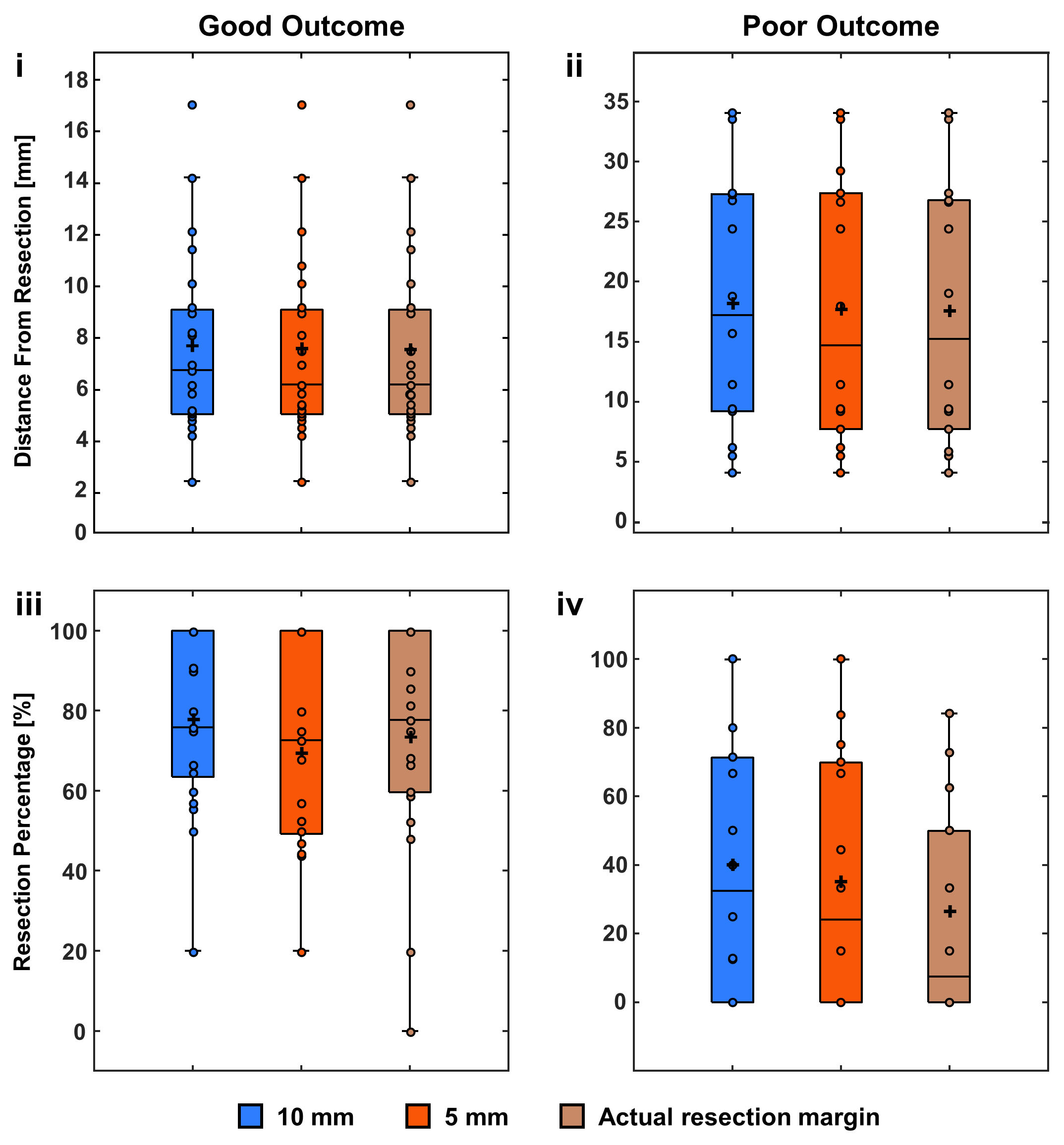


**Supplementary Figure S2. Distance from resection and resection percentage of the spike-ripple onset overlap zone (SRO) at different resection cut-off thresholds (10 mm, 5 mm, and using the actual resection margins).** **(i)** Comparing the distance of SRO from the resection zone at 10 mm, 5 mm, and by using the actual resection margins in good outcome patients (Engel I, 21 patients). **(ii)** Comparing the distance of SRO from the resection zone at 10 mm, 5 mm, and by using the actual resection margins in poor outcome patients (Engel ≥II, 14 patients). **(iii)** Comparing the overlap with resection for SRO at 10 mm, 5 mm, and by using the actual resection margins in good outcome patients. **(iv)** Comparing the overlap with resection for SRO at 10 mm, 5 mm, and by using the actual resection margins in poor outcome patients. In the boxplots, the cross indicates the mean value, and the horizontal lines indicate the median value, lower and upper edges represent the 25th and 75th percentiles, whiskers extend to the 0th and 100th percentiles (excluding outliers) and points outside the whiskers represent the outliers (i.e., values that are at least 1.5 times the interquartile range below the 25th percentile or above the 75th percentile). The multiple comparisons issue was accounted for using the false discovery rate (FDR) correction.
