# Supplementary material for: Overlap of spike and ripple propagation onset predicts surgical outcome in epilepsy
Source: Ann Clin Transl Neurol. 2024 Oct 7;11(10):2530–47. doi: 10.1002/acn3.52156 (PMC11514932; doi:10.1002/acn3.52156)
Supplement: Supplementary file 3 — Figure S3. [file ACN3-11-2530-s001.docx]

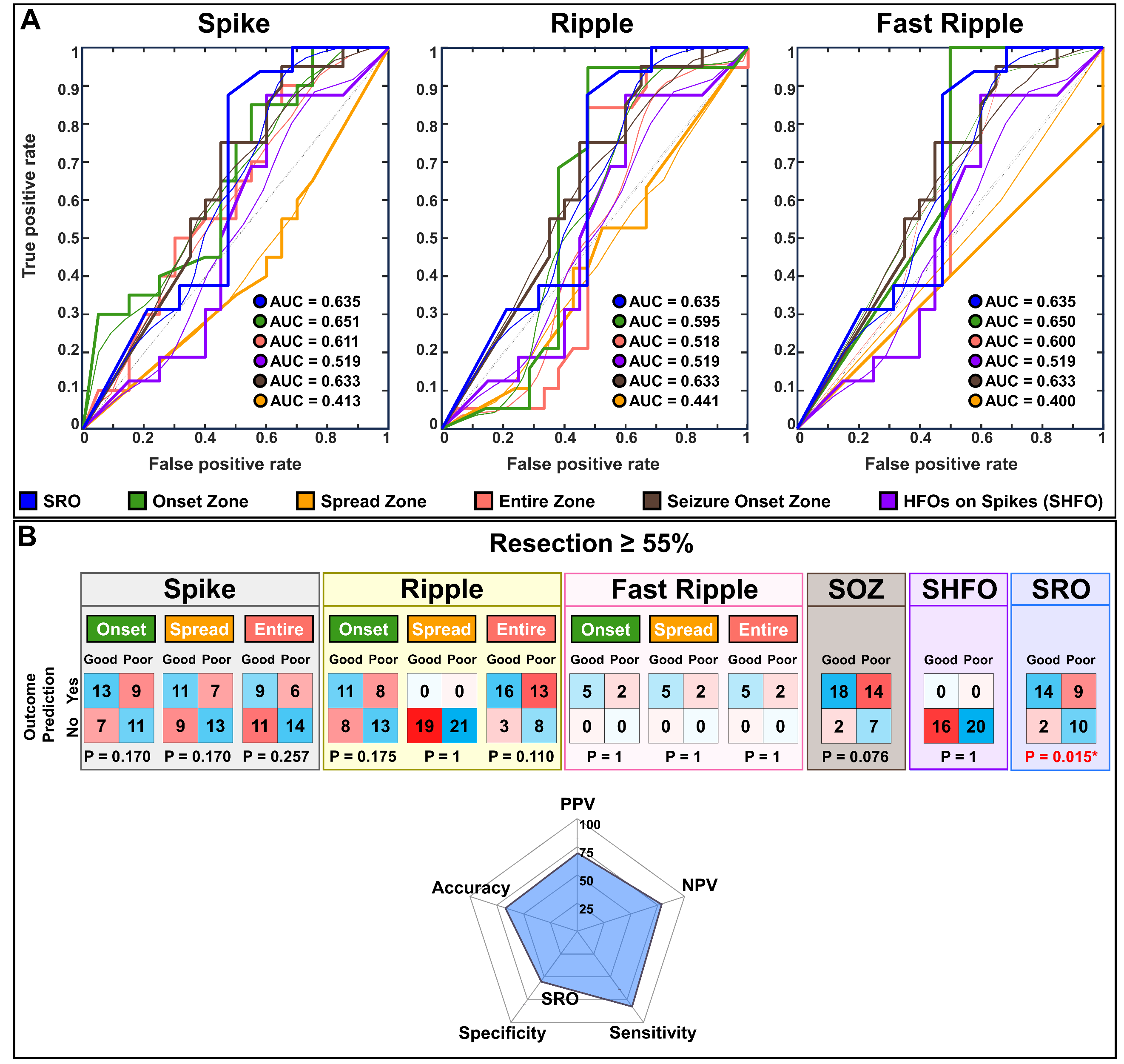


**Supplementary Figure S3. Outcome prediction results, considering Engel I-a as good outcome.** **(A)** From left to right: receiver operating characteristic (ROC) curve and their area under the curve (AUC) for onset, spread, and entire zones of spike (40 patients), ripple (40 patients), and fast ripple (seven patients) propagations, spike co-occurring with HFOs (SHFO) zone (36 patients), the spike-ripple onset overlap zone (SRO, 35 patients), and the seizure onset zone (SOZ, 41 patients) as predictors of post-surgical outcome. **(B)** Confusion matrix for each of the zones in predicting the surgical outcome at resection threshold of 55% using the logistic regression with leave one out cross validation method and the spider plot of positive predictive value (PPV), negative predictive value (NPV), sensitivity, specificity, and accuracy of each of the zones for predicting the outcome corresponding to the resection threshold of 55%.
