# Supplementary material for: Overlap of spike and ripple propagation onset predicts surgical outcome in epilepsy
Source: Ann Clin Transl Neurol. 2024 Oct 7;11(10):2530–47. doi: 10.1002/acn3.52156 (PMC11514932; doi:10.1002/acn3.52156)
Supplement: Supplementary file 4 — Table S1. [file ACN3-11-2530-s004.docx]

**Supplementary Table S1. Sensitivity and Precision of Spike Detections and Percentage of rejected HFOs**

| **ID** | **Spike Detections** | | **HFO Detections** | |  |
| --- | --- | --- | --- | --- | --- |
|  | **Sensitivity**  **[%]** | **Precision**  **[%]** | **Percentage of rejected ripples [%]** | **Percentage of rejected fast ripples [%]** | **Sampling Frequency**  **[Hz]** |
| 3 | 85 | 67 | 39 | 0 | 2048 |
| 8 | 41 | 53 | 94 | 20 | 2000 |
| 14 | 50 | 72 | 36 | 2 | 2000 |
| 20 | 91 | 93 | 48 | 45 | 2048 |
| 23 | 97 | 81 | 33 | 8 | 2048 |
