# Supplementary material for: Overlap of spike and ripple propagation onset predicts surgical outcome in epilepsy
Source: Ann Clin Transl Neurol. 2024 Oct 7;11(10):2530–47. doi: 10.1002/acn3.52156 (PMC11514932; doi:10.1002/acn3.52156)
Supplement: Supplementary file 5 — Table S2. [file ACN3-11-2530-s003.docx]

**Supplementary Table S2. Postsurgical Outcome Prediction Values of Different Zones: Logistic Regression with Leave One Out Cross Validation**

| **Res.** | **Spikes** | | | | **Ripples** | | | | **Fast Ripples** | | |  |  |  |
| --- | --- | --- | --- | --- | --- | --- | --- | --- | --- | --- | --- | --- | --- | --- |
| **>50%** | **Ons.** | **Spr.** | **Ent.** | **Ons.** | | **Spr.** | **Ent.** | **Ons.** | | **Spr.** | **Ent.** | **SOZ** | **sHFO** | **SRO** |
| PPV | 76.9 | 62.5 | 78.9 | 79.2 | | 46.7 | 60.0 | 71.4 | | 71.4 | 71.4 | 68.6 | 72.7 | 79.2 |
| NPV | 64.3 | - | 52.4 | 68.8 | | 0 | - | - | | - | - | 83.3 | 64.3 | 81.8 |
| SENS | 80.0 | 100 | 60.0 | 79.2 | | 58.3 | 100 | 100 | | 100 | 100 | 96.0 | 76.2 | 90.5 |
| SPEC | 60.0 | 0 | 73.3 | 68.8 | | 0 | 0 | 0 | | 0 | 0 | 31.3 | 60.0 | 64.3 |
| ACC | 72.5 | 62.5 | 65.0 | 75.0 | | 35.0 | 60 | 71.4 | | 71.4 | 71.4 | 70.7 | 69.4 | 80.0 |
| ***P***^a^ | 0.01^*^ | 1 | 0.04^*^ | <0.01^*^ | | 1 | 1 | 1 | | 1 | 0.29 | 0.03^*^ | 0.03^*^ | <0.01^*^ |
| **>55%** | **Ons.** | **Spr.** | **Ent.** | **Ons.** | | **Spr.** | **Ent.** | **Ons.** | | **Spr.** | **Ent.** | **SOZ** | **sHFO** | **SRO** |
| PPV | 81.8 | 62.5 | 59.1 | 80.0 | | 44.8 | 60.0 | 71.4 | | 71.4 | 71.4 | 74.2 | 47.8 | 79.2 |
| NPV | 61.1 | - | 33.3 | 60.0 | | 0 | - | - | | - | - | 77.8 | 23.1 | 76.9 |
| SENS | 72.0 | 100 | 52.0 | 66.7 | | 54.2 | 100 | 100 | | 100 | 100 | 92.0 | 52.4 | 86.4 |
| SPEC | 73.3 | 0 | 40.0 | 75.0 | | 0 | 0 | 0 | | 0 | 0 | 46.7 | 20.0 | 66.7 |
| ACC | 72.5 | 62.5 | 47.5 | 70.0 | | 32.5 | 60 | 71.4 | | 71.4 | 71.4 | 75 | 38.9 | 78.4 |
| ***P***^a^ | <0.01^*^ | 1 | 0.7 | 0.01^*^ | | 1 | 1 | 1 | | 1 | 1 | <0.01^*^ | 0.9 | <0.01^*^ |
| **>60%** | **Ons.** | **Spr.** | **Ent.** | **Ons.** | | **Spr.** | **Ent.** | **Ons.** | | **Spr.** | **Ent.** | **SOZ** | **sHFO** | **SRO** |
| PPV | 80.1 | 62.5 | 62.5 | 52.0 | | 44.8 | 60.0 | 66.7 | | 71.4 | 60.0 | 74.2 | 40.0 | 77.3 |
| NPV | 57.9 | - | - | 26.7 | | 0 | - | 0 | | - | 0 | 77.8 | 0 | 66.7 |
| SENS | 68.0 | 100 | 100 | 54.2 | | 54.2 | 100 | 80.0 | | 100 | 60.0 | 92.0 | 47.6 | 77.3 |
| SPEC | 73.3 | 0 | 0 | 25.0 | | 0 | 0 | 0 | | 0 | 0 | 46.7 | 0 | 66.7 |
| ACC | 70.0 | 62.5 | 62.5 | 42.5 | | 32.5 | 60 | 57.1 | | 71.4 | 42.9 | 75.0 | 27.8 | 73.0 |
| ***P***^a^ | 0.01^*^ | 1 | 1 | 0.9 | | 1 | 1 | 1 | | 1 | 1 | <0.01^*^ | 1 | <0.01^*^ |
| **>65%** | **Ons.** | **Spr.** | **Ent.** | **Ons.** | | **Spr.** | **Ent.** | **Ons.** | | **Spr.** | **Ent.** | **SOZ** | **sHFO** | **SRO** |
| PPV | 51.6 | 62.5 | 62.5 | 40.1 | | 42.9 | 55.6 | 66.7 | | 71.4 | 60.0 | 79.2 | 52.6 | 75.0 |
| NPV | 0 | - | - | 0 | | 0 | 0 | 0 | | - | 0 | 62.5 | 35.3 | 58.8 |
| SENS | 64 | 100 | 100 | 45.8 | | 50.0 | 83.3 | 80.0 | | 100 | 60.0 | 76.0 | 47.6 | 68.2 |
| SPEC | 0 | 0 | 0 | 0 | | 0 | 0 | 0 | | 0 | 0 | 66.7 | 40.0 | 66.7 |
| ACC | 40.0 | 62.5 | 62.5 | 27.5 | | 30.0 | 50.0 | 57.1 | | 71.4 | 42.9 | 72.5 | 44.4 | 67.6 |
| ***P***^a^ | 1 | 1 | 1 | 1 | | 1 | 1 | 1 | | 1 | 1 | <0.01^*^ | 0.8 | 0.04^*^ |
| **>70%** | **Ons.** | **Spr.** | **Ent.** | **Ons.** | | **Spr.** | **Ent.** | **Ons.** | | **Spr.** | **Ent.** | **SOZ** | **sHFO** | **SRO** |
| PPV | 62.5 | 62.5 | 62.5 | 38.5 | | 60.0 | 55.6 | 66.7 | | 71.4 | 71.4 | 77.3 | 58.3 | 77.8 |
| NPV | - | - | - | 0 | | - | 0 | 0 | | - | - | 55.6 | - | 57.9 |
| SENS | 100 | 100 | 100 | 41.7 | | 100 | 83.3 | 80 | | 100 | 100 | 68.0 | 100 | 63.6 |
| SPEC | 0 | 0 | 0 | 0 | | 0 | 0 | 0 | | 0 | 0 | 66.7 | 0 | 73.3 |
| ACC | 62.5 | 62.5 | 62.5 | 25.0 | | 60 | 50.0 | 57.1 | | 71.4 | 71.4 | 67.5 | 58.33 | 67.6 |
| ***P***^a^ | 1 | 1 | 1 | 1 | | 1 | 1 | 1 | | 1 | 1 | 0.04^*^ | 1 | 0.03^*^ |
| **>75%** | **Ons.** | **Spr.** | **Ent.** | **Ons.** | | **Spr.** | **Ent.** | **Ons.** | | **Spr.** | **Ent.** | **SOZ** | **sHFO** | **SRO** |
| PPV | 62.5 | 62.5 | 59.5 | 60.0 | | 60.0 | 60.0 | 66.7 | | 71.4 | 71.4 | 48.4 | 58.3 | 82.4 |
| NPV | - | - | 0 | - | | - | - | 0 | | - | - | 0 | - | 61.1 |
| SENS | 100 | 100 | 88 | 100 | | 100 | 100 | 80 | | 100 | 100 | 60.0 | 100 | 66.7 |
| SPEC | 0 | 0 | 0 | 0 | | 0 | 0 | 0 | | 0 | 0 | 0 | 0 | 78.6 |
| ACC | 62.5 | 62.5 | 55.0 | 60 | | 60 | 60 | 57.1 | | 71.4 | 71.4 | 36.6 | 58.3 | 71.4 |
| ***P***^a^ | 1 | 1 | 1 | 1 | | 1 | 1 | 1 | | 1 | 1 | 1 | 1 | 0.01^*^ |

ACC = Accuracy; Ent = Entire; NPV = Negative Predictive Value; Ons = Onset; PPV = Positive Predictive Value; Res = Resection; SENS = Sensitivity; sHFO = spikes cooccurring with HFOs; SOZ = Seizure Ons. Zone; SPEC = Specificity; Spr = Spread; SRO = Spike-Ripple Onset overlap.

^*^Significant Fisher’s exact test (P < 0.05)

^a^Fisher’s exact test.
