# Supplementary material for: Overlap of spike and ripple propagation onset predicts surgical outcome in epilepsy
Source: Ann Clin Transl Neurol. 2024 Oct 7;11(10):2530–47. doi: 10.1002/acn3.52156 (PMC11514932; doi:10.1002/acn3.52156)
Supplement: Supplementary file 6 — Table S3. [file ACN3-11-2530-s006.docx]

**Supplementary Table S3. Fast ripple detection in different durations of iEEG recordings**

| **ID/**  **Engel** | **channels in propagations**  **[No.]** | **Rate of Fast ripples per channel**  **[No./ min]** | **Rate of Fast ripple propagations [No./min]** | **O_res_**  **[%]** | **D_res_**  **[mm]** | **Sampling Frequency**  **[Hz]** |
| --- | --- | --- | --- | --- | --- | --- |
| **Epoch Duration = 300 s** | | | | | | |
| 1**/**IA | **12** | 1.5 | 15.8 | 67 | 10.7 | 2000 |
| 7/IA | **0** | 0.4 | **0** | 67 | 5.7 | 2000 |
| 8/IB | 0 | 0.2 | 0 | 88 | 9.2 | 2000 |
| 28/IIIA | 51 | 3.8 | 40.2 | 24 | 28.8 | 2000 |
| 35/IIB | 0 | 0.9 | **0** | 83 | 10.6 | 2000 |
| **Epoch Duration = 600 s** | | | | | | |
| 1**/**IA | 15 | 1.6 | 18.3 | 55 | 15.6 | 2000 |
| 7/IA | 0 | 0. 3 | 0 | 67 | 5.7 | 2000 |
| 8/IB | 0 | 0.1 | 0 | 88 | 9.2 | 2000 |
| 28/IIIA | 44 | 2.8 | 37.1 | 27 | 27.6 | 2000 |
| 35/IIB | 0 | 0.4 | 0 | 86 | 9.7 | 2000 |
| **Epoch Duration = 1200 s** | | | | | | |
| 1**/**IA | 17 | 1.3 | 21.6 | 51 | 15.9 | 2000 |
| 7/IA | 0 | 0.3 | 0 | 67 | 5.7 | 2000 |
| 8/IB | 0 | 0.1 | 0 | 48 | 14.8 | 2000 |
| 28/IIIA | 43 | 2.9 | 37.8 | 27 | 27.2 | 2000 |
| 35/IIB | 0 | 0.2 | 0 | 86 | 9.7 | 2000 |
| **Epoch Duration = 1800 s** | | | | | | |
| 1**/**IA (1) | 16 | 1.0 | 22.4 | 51 | 15.9 | 2000 |
| 7/IA (12) | 0 | 0.3 | 0 | 67 | 5.7 | 2000 |
| 8/IB (13) | 0 | 0.1 | 0 | 46 | 15.0 | 2000 |
| 28/IIIA (18) | 43 | 2.7 | 37.3 | 27 | 27.2 | 2000 |
| 35/IIB (37) | 0 | 0.1 | 0 | 86 | 9.7 | 2000 |

No. = Number; D = Average distance of fast ripple zone from resection; res = resection; O = Overlap.
