# Supplementary material for: Overlap of spike and ripple propagation onset predicts surgical outcome in epilepsy
Source: Ann Clin Transl Neurol. 2024 Oct 7;11(10):2530–47. doi: 10.1002/acn3.52156 (PMC11514932; doi:10.1002/acn3.52156)
Supplement: Supplementary file 7 — Data S1. [file ACN3-11-2530-s007.docx]

**Supplementary Material**

## Automatic Detection and Visual Inspection of Spikes and HFOs

The first step of interictal spikes detection was performed automatically using *Persyst 14.0* (Persyst Development Co., CA)^41^ in the frequency band of 1-70 Hz and by setting the detection sensitivity to medium (50%). The automatic spike detections were further evaluated against visually marked spikes for five random patients, performed by (C.P.) who was blind to the automatic spike detections. We considered a spike event from *Persyst* as a true positive (TP) if the event was confirmed by the visual inspection, and as a false positive (FP) if the event was rejected in the visual inspection. Also, spike events that were marked in the visual inspection but were missed by *Persyst* were counted as a false negative (FN). For each of these five patients, we calculated sensitivity [TP/(TP$+$FN)] and precision [TP/(TP$+$FP)] of *Persyst* in detecting interictal spikes [median sensitivity 85 (48-53) % and median precision 72 (64-84) %, Supplementary Table S1].

The first step of HFO detection was performed using RippleLab^42^ by breaking the iEEG data into 60 second windows in the frequency bands of 80-250 Hz for ripple and 250-500 Hz for fast ripple detection. A candidate HFO was initially selected if it had a minimum length of 10 ms and contained a local maximum of at least five standard deviations greater than the envelope of the signal obtained by Hilbert Transform. To further enhance the robustness of HFO detection, we performed additional analysis by plotting the time-frequency plane for each HFO and visually inspecting (L.F.) to ensure the inclusion of an actual HFO and not a filtering effect. The inspection fulfilled the following criteria: a ripple or fast ripple event was regarded as a true event if showing an isolated peak (island) in the time-frequency plane.^4,26^ Contrarily, ripple and fast ripple events that showed sparse islands or elongated islands within the time interval of the event were regarded as artifacts and excluded from further analysis.^26^ We calculated the percentage of rejected ripples and fast ripples for five random patients [ripples: 39 (35-60)%, fast ripples: 8 (2-26)% , Supplementary Table S1].

## Electrodes Ranking

For each propagation type, electrodes that contained event counts <20% of the maximum count among electrodes were regarded as inactive and omitted from ranking. To remove outliers, propagations having >50% of their events occurring within 2 ms from each other were discarded.^44,45^ We also discarded propagations that included >75% of the total electrodes number were excluded from further analysis. The 75% threshold for outlier propagations was chosen as the third quartile in a normal distribution and based on the premise that propagations engaging high percentages of electrodes are more likely indicative of widespread, non-localized brain activity rather than specific focal patterns. In order to rank the electrodes in a propagation, a score of one was given to the first electrode in the propagation and the score incremented by one for the next electrode in the sequence. Using these scores for each propagation type, we estimated the electrode rank (*ER*) for each electrode as the median of all scores that the electrode obtained. *ER*s were finally normalized based on the electrode with the maximum *ER* for each patient, representing the overall temporal activity of each electrode in propagation activities, using the following equation:

$$ER_{normalized}=1-\frac{ER-min(ER)}{max(ER)-min(ER)}$$

## Analysis of Fast Ripples in Longer Data Segments

We further investigated whether the duration of iEEG data affected the distance from resection and overlap with resection of the fast ripple zone. We analyzed longer iEEG recordings, specifically 600, 1200, and 1800 s, in five random patients [two good (Engel I) and three poor (Engel ≥I) outcomes]. We then calculated the fast ripples rate and their propagation rate, the number of channels that recorded fast ripples and their overlap with resection, and their average distance from resection in these extended iEEG recordings (Supplementary Table S3). Comparisons between the distances of the electrodes in the entire fast ripple zone from resection in different iEEG data durations did not show significant differences (*p* > 0.05).

**Defining Resected Electrodes**

In this study, the resection was defined by co-registering pre- and post-surgical MRIs. Each electrode was regarded as resected if it was inside or within 10 mm from resection; otherwise, it was considered non-resected. The 10 mm cut-off was based on previous studies indicating that the mean gyral width ranges from 11 to 21 mm^38,39^. Based on these previous studies, we presumed that an electrode within 10 mm of the resection margin would belong to the same gyrus.

To assess the sensitivity of our results to this cut-off, we compared the resection percentage and the distance from resection of SRO, separately for good and poor outcome patients, at three different cut-off values: 10 mm, 5 mm, and considering electrodes as resected only if they were within the resection volume. We did not observe any significant differences (Supplementary Figure S2).

## Considering Engel I-a as Good Outcome

We reevaluated the best thresholds for defining the onset zones of spike, ripple, and fast ripple propagations by only considering patients with surgery outcome of Engel I-a as good outcome patients and studied epileptogenic zone and outcome predictability of the different zones (Supplementary Figures S1 and S3).

## Considering Engel I-a as Good Outcome: Distance From Resection and Resection Percentage of different zones

We provide statistics of distance from resection and resection percentage as mean ± standard deviation. In good outcome patients (Engel I-a), SRO was closer to resection (8.2 ± 3.8 mm) compared to SHFO zone (12.2 ± 5.9 mm, *p =* 0.023) and SOZ was closer to resection (6.8 ± 2.9 mm) compared to the ripple onset zone (11.9 ± 3.8 mm, *p =* 0.003). Spike onset zone had a distance of (11.5 ± 6.8) [Supplementary Figure S1(i)]. The distance of spike onset was shorter in good (11.1 ± 6.8 mm) compared to poor outcome patients (17.3 ± 10.0 mm, p = 0.033). There was no difference in distances of ripple, and fast ripple onsets, as well as SHFO and SRO to resection for good vs. poor outcome patients [Supplementary Figure S1(ii)].

In good outcome patients, average resection was higher for SRO (75 ± 23%) and SOZ (82 ± 20%) compared to ripple (61 ± 14%, *p =* 0.023 and *p =* 0.004 respectively) onset. No differences were observed for resection between the other zones [Supplementary Figure S1(iii)]. There was no difference in resection of spike, ripple, and fast ripple onsets, as well as SHFO and SRO in good vs. poor outcome patients [Supplementary Figure S1(iv)].

## Considering Engel I-a as Good Outcome: Outcome Prediction

The AUC of SRO for predicting outcome was comparable to the AUC of the SOZ, and onset zones of spikes and fast ripples (Supplementary Figure S3A). By considering resection threshold of 55%, only resection of SRO predicted outcome (*p =* 0.022), with a positive predictive value of 61% and negative predictive value of 79% (Supplementary Figure S3B).
